# Thermal Instability Pathways of Aluminum‐Based Nanocomposites: The Role of Alloying‐Element‐Driven Precipitation and Amorphous Interlayers

**DOI:** 10.1002/smll.75393

**Published:** 2026-08-22

**Authors:** Hendrik C. Jansen, Amit Sharma, Marcus Hans, Fedor F. Klimashin, Léo Lapeyre, Dominik Gutnik, Jochen M. Schneider, Thomas E.J. Edwards, Jakob Schwiedrzik, Barbara Putz, Johann Michler

**Affiliations:** ^1^ Laboratory For Mechanics of Materials and Nanostructures Empa Swiss Federal Laboratories for Materials Science and Technology Thun Switzerland; ^2^ École Polytechnique Fédérale de Lausanne (EPFL) Lausanne Switzerland; ^3^ Materials Chemistry RWTH Aachen University Aachen Germany; ^4^ Department of Materials Science University of Leoben Leoben Austria; ^5^ Research Center for Structural Materials National Institute for Materials Science Tsukuba Japan; ^6^ Department of Materials Science Institute of Pure and Applied Sciences University of Tsukuba Tsukuba Ibaraki Japan; ^7^ Laboratory For High Performance Ceramics Empa Swiss Federal Laboratories for Material Science and Technology Dübendorf Switzerland

**Keywords:** hybrid PVD / ALD, nanocrystalline Al, nanoindentation, phase transformation, thermal stability

## Abstract

Hybrid physical vapour deposition (PVD) combined with atomic layer deposition (ALD) enables the fabrication of metal/ceramic AlNi/AlO(H) nanolaminates with high room‐temperature strength, controlled architecture, and promising functional performance, yet their thermal stability remains unresolved. Here, we establish design principles for thermally robust Al‐based nanocomposites by systematically assessing their thermal instability through correlated synchrotron X‐ray diffraction, high‐resolution microscopy, atom probe tomography, and nanoindentation following vacuum annealing between 160°C and 600°C. Angle‐resolved X‐ray diffraction (XRD) reveals Al crystallite evolution while high‐resolution microscopy unravels further instability mechanisms: At homologous temperature *T_hom_* ≈ 0.52 (∼210°C), Ni segregation to Al grain boundaries competes with Al_3_Ni formation. Hydroxylated ALD‐AlO*_x_* H*_y_* interlayers become mobile, driving swelling and oxidation of adjacent Al. At *T_hom_* ≈ 0.77 (∼450°C), *κ*‐Al_2_O_3_ crystallises, followed by interlayer rupture, while at *T_hom_* ≈ 0.94 (∼600°C) the nanolaminate architecture collapses into a coarsened Al(Ni) matrix with dispersed Al_2_O_3_ inclusions. These coupled transformations progressively reduce hardness, transitioning from confined layer slip to matrix–inclusion‐dominated deformation. Beyond elucidating degradation mechanisms, this work shows how hydrogen‐mediated interface dynamics and the dual role of alloyed‐Ni limit the thermal and mechanical stability, precisely identifying the onset temperatures, and providing a pathway to robust Al‐based PVD/ALD nanocomposites.

## Graphical abstract

Thermal instability mechanisms governing novel Al‐based PVD/ALD nanocomposites are uncovered through synchrotron XRD, STEM, APT, and micromechanical testing. Temperature‐dependent crystallite evolution reveals a defined stability window before degradation dominates, offering new insight into the design of thermally robust, functional Al‐based nanocomposites.

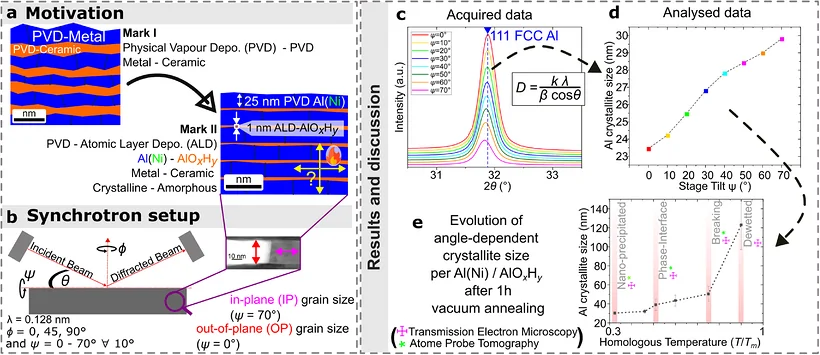

## 1 Introduction

Nanocrystalline Al offers exceptional strength and low density, but suffers from limited thermal stability (∼200°C) as rapid grain coarsening at elevated temperatures leads to a loss of strength [1]. The driving force for grain growth in Al is particularly high due to its low melting point and high diffusivity [2], which renders its nanostructure thermodynamically unstable even at moderate homologous temperatures (*T_hom_* = *T*/*T_m_*; where *T* and *T_m_* are the operating and melting temperature and both in unit of Kelvin). In recent years, considerable effort has therefore been devoted to enhancing the thermal stability of nanocrystalline Al through segregation engineering, either by forming nanoscale precipitates that provide Zener pinning, or introducing solute atoms that segregate to grain boundaries to reduce their energy and mobility [3]. Kinetic stabilisation in nanocrystalline Al was reported by Huang, Kalidindi, and Schuh [4] for electrodeposited Al‐6.5 at.% Mn, maintaining sub‐100 nm grain size after *T_hom_ =* 0.61 (∼300°C) annealing in inert atmosphere for up to 1 month through the formation of Al_6_Mn nanoprecipitates. Similarly, nanoscale Al_3_Mg_2_ precipitates were found to kinetically stabilise magnetron‐sputtered Al‐5 and Al‐10 at.% Mg films, which maintained sub‐10 nm grain size after *T_hom_ =* 0.61 (∼300°C) vacuum‐annealing for 3 h [5]. In a ball milled Al_90_Zr_10_ alloy, L1_2_‐Al_3_Zr nanoprecipitates were found to kinetically stabilise Al grain growth to only 58 nm after annealing at *T_hom_ =* 0.88 (∼550°C) for 1 h [6]. Gianola et al. [7] reported that grain boundary stabilisation toward higher yield strength in the sub‐100 nm range can already be achieved through ∼10 at.% O impurities in magnetron‐sputtered nanocrystalline Al upon base pressure variations (10^−7^ to 10^−5 ^mbar). In another study, intentional co‐alloying in the sputtering process gave rise to a binary 4‐nm columnar Al_94.5_Fe_5.5_ with thermal stability up to *T_hom_ =* 0.51 (∼200°C) with 1.3 GPa flow stress [8]. In summary, kinetic pinning by precipitates arised only at low to moderate homologous temperatures of *T_hom_* = 0.61 (∼300°C) and was limited by rapid coarsening at higher temperatures, leaving a narrow and fragile window of benefit. Enhanced thermodynamic stabilisation of grain boundaries by complexions has however been reported through chemical segregation of solutes that effectively lower the grain boundary energy and reduce its driving force for grain coalescence upon annealing [9, 10]. Yet, elements that refine Al grains may still induce deleterious segregation in the matrix and fail to enhance thermal resilience [11, 12]. Gianola and colleagues [11, 13, 14, 15, 16, 17] pioneered studies on the thermodynamic stabilisation of ternary nanocrystalline Al‐Mg‐Y, Al‐Fe‐Y, Al‐Ni‐Y, and Al‐Ni‐Ce alloys. Recently, a magnetron‐sputtered Al_94.9_Ni_3.8_Ce_1.3_ was able to maintain sub‐10 nm Al grain size upon annealing at *T_hom_* = 0.64 (∼325°C) for 1 h, while only above *T_hom_* = 0.70 (∼380°C) Al grain coarsening did occur [16]. Notably, the stabilisation of nanocrystalline Al was correlated to the formation of NiCe‐rich amorphous intergranular films, while intermetallic Al_3_Ni and Al_11_Ce_3_ phases precipitated above *T_hom_* = 0.70 (∼380°C). The additions of Ni and Ce were therefore able to prevent previously reported softening at *T_hom_* = 0.42 (∼120°C) for nanocrystalline Al_95.5_Ni_4.5_ [18] and recrystallisation at *T_hom_* = 0.53 (∼220°C) for amorphous Al‐Ni [19]. Notably, magnetron sputtering imposes beneficial ultrahigh cooling rates up to 10^15^ K s^−1^ [20], which allow a high degree of disorder similar to the design of thin film metallic glasses [21, 22]. However, grain boundary stabilisation was also observed for ball‐milled Al_95.7_Mg_2.1_Y_2.2_, Al_95.7_Fe_2.2_Y_2.1_, and Al_95.7_Ni_2.2_Y_2.1_ that were able to maintain sub‐50 nm Al grain size up to *T_hom_* = 0.87 (∼540°C) for 1 h through a combination of amorphous Ni‐Y complexion and amorphous Ni‐Y core‐shell nanorod formation at grain boundaries [11].

In contrast to such chemical modification, structural leverage through systematic control over layer thickness and architecture can enable improvements in both strength [23] and eventually, thermal stability [24]. While nanolaminating Al with e.g., AlN [25], SiC [26], crystalline Al_2_O_3_ [27], and amorphous Al_2_O_3_ [28, 29, 30] has been widely studied to enhance mechanical properties, the literature lacks studies on similar effects for enhanced thermal stability. Notably, enhanced thermal stability through nanolaminating was not yet reported for Al and mainly investigated in crystalline / crystalline Cu / Nb, where the combination of face centered cubic (FCC) Cu and body centered cubic (BCC) Nb enhanced the poor thermal stability of the FCC Cu through 3D interfaces up to 300°C for 1 h [31]. The combination of crystalline metallic and amorphous ceramic layers provides further opportunities, since e.g., amorphous Al_2_O_3_ exhibits higher thermal stability than FCC Al and does not crystallise below 1000°C [32]. Accordingly, the out‐of‐plane (OP) grain growth of the nanocrystalline layer is impeded, up to the stability limit of the amorphous interlayer. We have recently applied the interface‐engineered design strategy of hybrid physical vapour deposition (PVD) and atomic layer deposition (ALD) to Al_100‐_ *_z_* Ni*_z_* / AlO*_x_* H*_y_* (25 / 1 nm) [33], exploiting OP control by AlO*_x_* H*_y_* interlayers and in‐plane (IP) control by chemical complexity through Al_100‐_ *_z_* Ni*_z_*. In situ transmission electron microscopy (TEM) has already unravelled the high thermal stability of thin 4‐nm ALD‐Al_2_O_3_ layers up to 900°C [34], which is significantly higher than the eutectoid temperature of Al‐Ni around 640°C [35]. Here, ultimately, we combine both chemical and structural leverage toward Al_100‐_ *_z_* Ni*_z_* / AlO*_x_* H*_y_* (25 / 1 nm) thin films in a novel way and approach their thermomechanical stability.

## 2 Results and Discussion

### 2.1 Elucidating Thermal Stability

Crystallite size evolution of (111) FCC Al after vacuum annealing serves as an initial indicator of thermal instability in the nanolaminates, providing a basis for broader analyses that will later include phase separation and decomposition effects. The results from synchrotron XRD are displayed in Figure 1. While Figure 1a shows the derived OP‐Al crystallite size (*ψ* = 0°) as a function of *T_hom_*, Figure 1b displays IP‐Al crystallite size (*ψ* = 70°) approximations. In comparison to well‐established crystallite size determination through dark field (DF)‐TEM, IP‐Al crystallite size derived from synchrotron XRD at *ψ* = 70° and DF‐TEM crystallite size correlate with R^2^ = 0.89 – justifying the fast screening technique especially for *T_hom_ *< 0.94 here. Representative DF‐TEM images and grain size correlation (DF‐TEM vs. Scherrer‐XRD) can be found in Section S1. Error bars in Figure 1 are derived from the quadrature rule by Pearson VII fit quality, instrument broadening corrected by a LaB_6_ standard at every *ψ*, and three different stage rotations *ϕ*.

**FIGURE 1:**
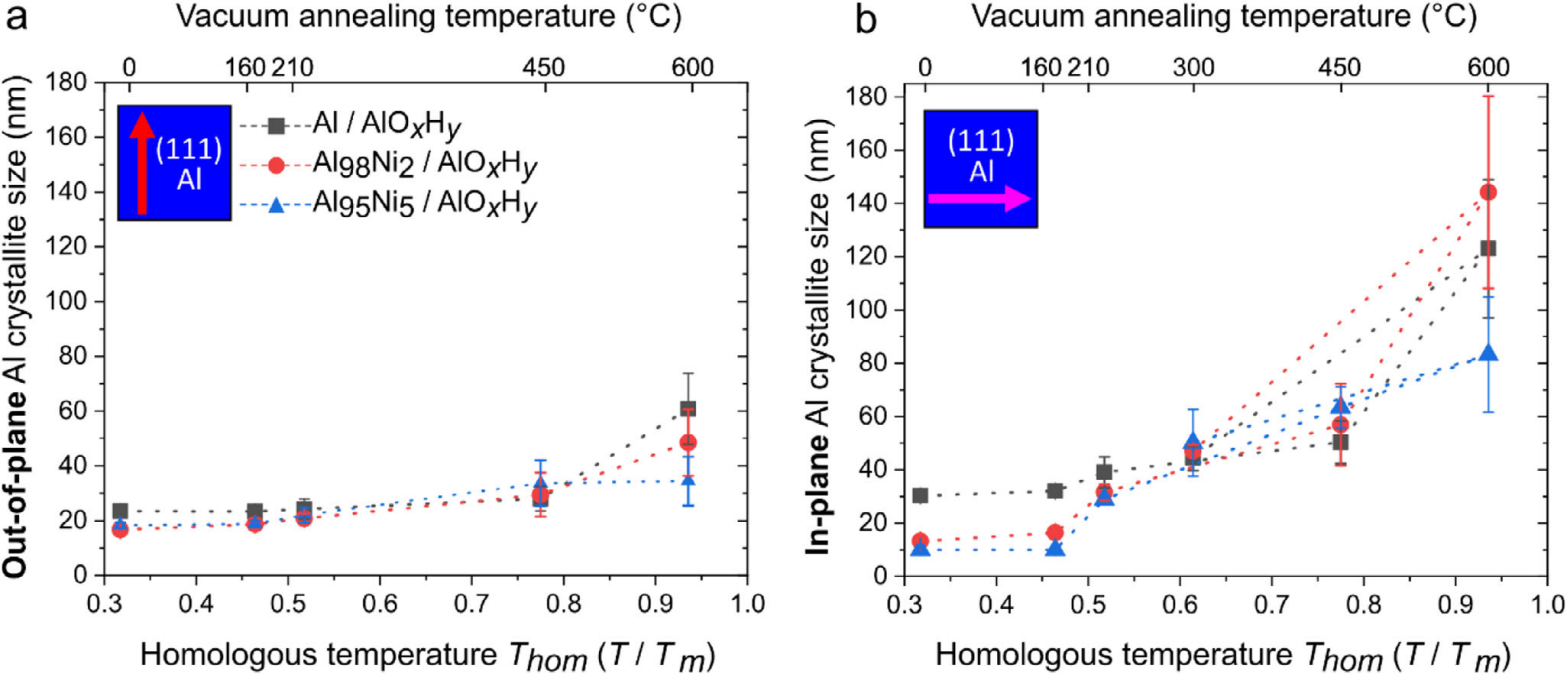
Evolution of Al microstructure over ex situ vacuum annealing for 1 h in Al_100‐_ *_z_* Ni*_z_*–AlO*_x_* H*_y_* thin films: (a) Out‐of‐plane crystallite and (b) in‐plane Al crystallite size as a function of homologous temperature. Note that the ordinate scales differ.

The OP‐Al crystallite size in Al – AlO*_x_* H*_y_* thin films stays constant around 25 ± 2 nm until *T_hom_ =* 0.77. Ni‐doped films show almost constant OP‐Al crystallite size only until *T_hom_ =* 0.52, with 19 ± 2 and 20 ± 2 nm for 2 and 5 at.% doping, respectively. At *T_hom_ =* 0.77, the OP‐Al crystallite size of Ni‐doped films exceeds the pure reference OP crystallite size with 30 ± 8 and 34 ± 8 nm for 2 and 5 at.% Ni, respectively. These values represent a relative OP grain growth of ∼20%, 76%, and 84% for 0, 2, and 5 at.% doped films from as‐deposited to *T_hom_ =* 0.77. At *T_hom_ =* 0.94, an OP‐Al grain growth until 61 ± 13 and 48 ± 12 nm can be detected for 0 and 2 at.% Ni containing films. Yet, Al_95_Ni_5_ – AlO*_x_* H*_y_* OP‐Al crystallite size at *T_hom_ =* 0.94 is only slightly increased to 34 ± 9 nm.

The IP‐Al crystallite size in Figure 1b evolves differently over temperature and chemistry. Until *T_hom_ =* 0.46, the IP‐Al crystallite size for all three samples stays constant with an IP‐Al crystallite size refinement upon Ni addition. Notably, the IP‐Al crystallite sizes until *T_hom_ =* 0.46 are 31 ± 2, 14.5 ± 1, and 10 ± 1 nm for 0, 2, and 5 at.% Ni, respectively. This agrees with our previously reported IP‐Al crystallite size refinement by Ni addition [33]. Further, until *T_hom_ =* 0.77, the IP‐Al crystallite size increases stepwise to 50 ± 8, 57 ± 15, and 50 ± 13 nm for 0, 2, and 5 at.% Ni. Similarly to the jump in OP‐Al crystallite size at *T_hom_ =* 0.94, the IP‐Al crystallite size reaches 123 ± 26, 144 ± 36, and 83 ± 22 nm for 0, 2, and 5 at.% Ni, respectively. Here, the IP crystallite size significantly exceeds the OP crystallite size for all three chemistries and OP/IP crystallite size ratios of ∼2.0, ∼3.0, and ∼2.4 are determined for 0, 2, and 5 at.% Ni. This is a first indicator that the AlO*_x_* H*_y_* interlayers serve as dominating coalescence barriers rather than any structural or chemical modification due to Ni addition in the crystalline Al layers.

Yet, several aspects require critical evaluation to assess the robustness of the data. In view of the commonly reported ∼100 nm upper applicability threshold of the Scherrer approach, the crystallite sizes obtained in this work fall securely within the reliable measurement range of the method for all OP crystallite sizes, but only until *T_hom_ =* 0.77 (∼450°C) for the IP determination. Only the undoped Al – AlO*_x_* H*_y_* shows almost constant OP‐Al crystallite size in the lower *T_hom_* range, whereas OP‐Al crystallite sizes in Ni‐doped films at lower *T_hom_* are below 25 nm metal layer thickness. In the Scherrer formula, a lower‐bound coherent domain size underestimation originates from the contribution of lattice distortions to the full width at half maximum (FWHM) and/or peak position. However, as the Scherrer formula does not include microstrain, it is commonly seen as a lower boundary value. There is no peak shift of the (111) Al visible for samples annealed until *T_hom_* = 0.46, but only peak broadening. Due to the constant layer thickness, the observed IP peak broadening cannot stem from grain refinement. Thus, the underestimation of OP crystallite size at lower *T_hom_* might be due to the presence of lattice strain from the Al‐Ni solid solution and formation of Ni‐rich nanoclusters (discussed in Section 2.3). XRD was performed in the vicinity of both (111) and (200) FCC Al peaks, but the strong (111) texture of the architecture here does not allow a comparable analysis of the (200) FCC Al diffraction. Hence, the Williamson and Hall [36] method is not applicable to determine the actual microstrain values. Similar peak broadening was reported for ball‐milled Mg‐3 at.% Zn alloys due the formation of a solid solution [37] and *γ'* nanoprecipitates broadening diffraction peaks of the *γ* matrix in Ni‐based superalloys [38], which is in agreement with the formation of Ni‐rich nanoclusters in the Ni‐containing as‐deposited films at *T_hom_* < 0.52 here. In summary, and although the classical Scherrer approach here neglects microstrain broadening, the good quantitative agreement (R^2^ = 0.89) obtained in the present fits with DF‐TEM demonstrates that a reliable size analysis can still be achieved even without explicit strain determination. Activation energy analysis of grain growth in Al_100−_ *_z_* Ni*_z _*/ AlO*_x_* H*_y_* was performed and correlated with literature reports on nanocrystalline Al; the findings are discussed in Section S2.

Huang, Kalidindi, and Schuh [4] reported enhanced thermal stability of electrodeposited nanocrystalline Al_93.5_Mn_6.5_ with as‐fabricated Al grain size of 66 ± 16 nm and Al grain growth toward 100 ± 40 nm for annealing at both *T_hom_* = 0.51 (∼200°C) and *T_hom_* = 0.61 (∼300°C) for 1 h. In comparison, Al_95_Ni_5_ / AlO*_x_* H*_y_* retains a more refined IP‐Al crystallite size of 29 ± 1 and 63 ± 8 nm for vacuum annealing at *T_hom_* = 0.52 (∼210°C) and *T_hom_* = 0.61 (∼300°C), respectively. However, magnetron‐sputtered AlNiCe with Al crystallite size below 10 nm was found to not coarsen during 1 h annealing up to *T_hom_* ∼0.64 (∼325°C) for 1 h due to the formation of Ni‐Ce‐rich amorphous interphases at Al‐Al grain boundaries [16]. The addition of low amounts of Ce (2 at.%) to low Ni amounts (2 at.%) resulted in an amorphous intermixing of Ni and Ce at the grain boundaries, which is thermodynamically more favourable than matrix segregation.

In addition to the Al crystallite size, microstructure evolution and phase formation was probed by lab‐scale XRD. Respective diffractograms (2*θ* range from 20 to 50°) are displayed in Figure 2. In the reference Al – AlO*_x_* H*_y_* thin film, the Al layers have a (111) and (200) texture, whereas at higher *T_hom_*, there is an indication of crystallisation of orthorhombic *κ*‐Al_2_O_3_ with only one peak at 2*θ* = 34.7° (Figure 2a). With increasing annealing temperature, the FCC Al peaks slightly shift to higher 2*θ* angles until *T_hom_* = 0.77, whereas annealing at *T_hom_* = 0.94 induces a peak shift to lower 2*θ* angles again. In XRD and upon thermal annealing, the Ni‐doped films show three major differences: a strong (111) Al texture and a slight shift of Al reflections to smaller 2*θ* angles in the as‐deposited state; the formation of orthorhombic Al_3_Ni; as well as an earlier and more pronounced crystallisation of orthorhombic *κ*‐Al_2_O_3_. Films containing 2 at.% Ni, Figure 2c, indicate mid‐range order (“amorphous hump”) formation toward *κ*‐Al_2_O_3_ and Al_3_Ni after 1 h of annealing at *T_hom_* = 0.77, whereas after annealing at *T_hom_* = 0.94, respective crystalline phase peaks occur. The addition of 2 and 5 at.% Ni to the Al layers leads to a phase separation from FCC Al → FCC Al + orthorhombic Al_3_Ni upon annealing, which are the thermodynamically stable phases [35]. The Al_3_Ni crystallisation onset temperature (from XRD) is reduced with increasing Ni content, from *T_hom_* ≤ 0.94 with 2 at.% Ni to *T_hom_* ≤ 0.61 with 5 at.% Ni.

**FIGURE 2:**
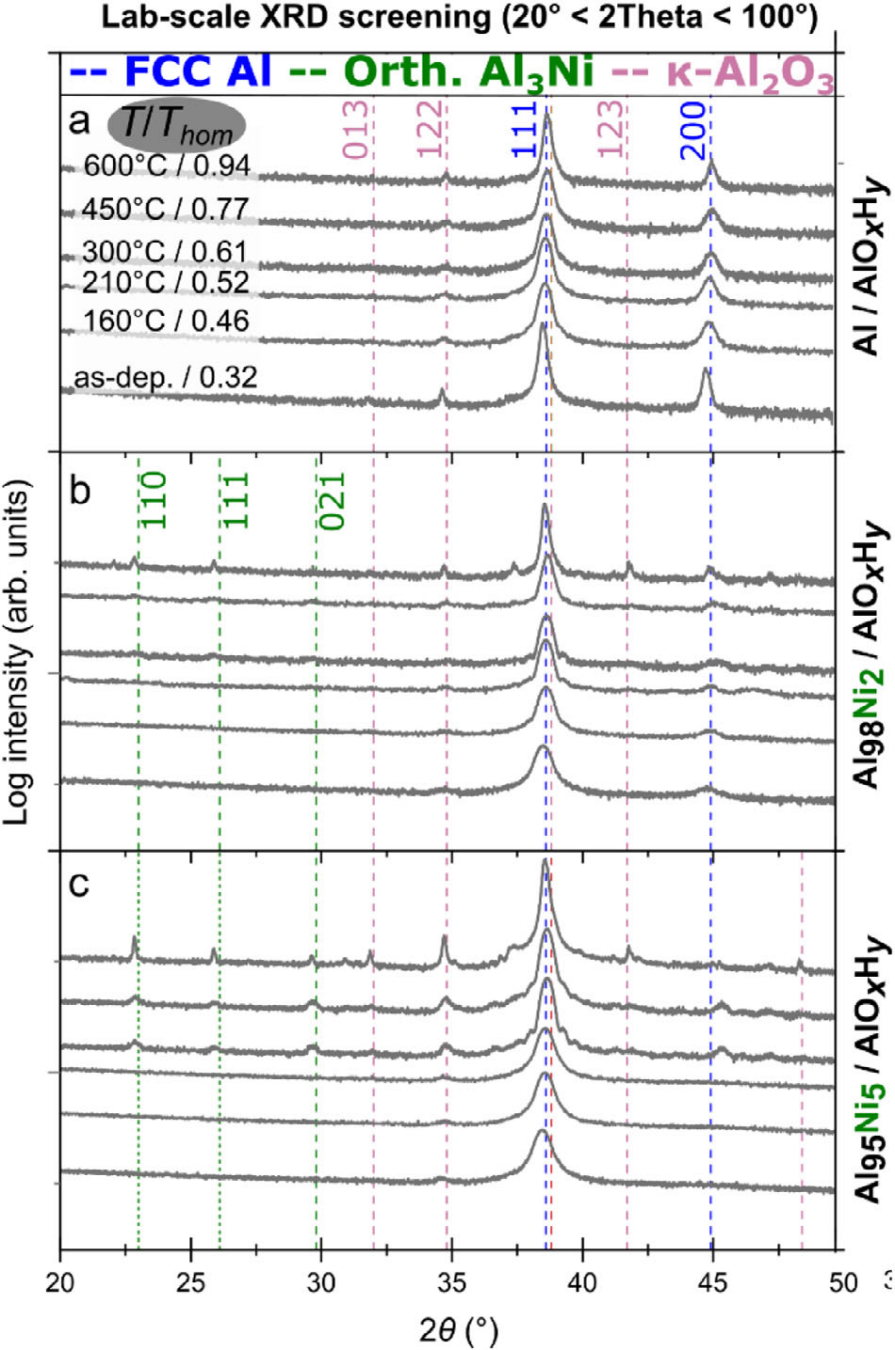
Diffraction data from lab‐scale: Evolution of phase formation in Al_100‐_ *_z_* Ni*_z_* / AlO*_x_* H*_y_* thin films probed by lab‐scale XRD in Bragg‐Brentano geometry of (a) Al / AlO*_x_* H*_y_*, (b) Al_98_Ni_2_ / AlO*_x_* H*_y_*, and (c) Al_95_Ni_5_ / AlO*_x_* H*_y_*.

The crystallisation of amorphous AlO*_x_* H*_y_* to metastable crystalline *κ*‐Al_2_O_3_ is seen as a destabilisation mechanism in the crystalline / amorphous microstructure. Here, three factors seem to influence the crystallisation: ultra‐thin 1 nm thickness, H‐enrichment through room temperature ALD, and Ni‐doping of the crystalline Al layers. Vogl et al. [34] observed the amorphous‐crystalline phase transition of ALD‐deposited amorphous 4 nm Al_2_O_3_ on Cu by in situ TEM at ∼1000°C. The ALD deposition temperature of 120°C in their study versus room‐temperature ALD here is expected to reduce the H concentration from > 20 at.% (thus AlO*_x_* H*_y_*) to < 10 at.% and approach higher density from < 2.4 to > 3 g/cm^3^ [39, 40]. Commonly, the crystallisation temperature of amorphous Al_2_O_3_ by ALD depends on the substrate and decreases with increasing annealing time and film thickness, while no crystallisation is usually observed at *T* < 700°C [32]. This hints toward H‐enrichment, rather than the 1 nm thickness of the AlO*_x_* H*_y_* interlayer, as a limiting factor for the integrity of the nanolaminated crystalline / amorphous architectures here, which will be discussed in chapter 2.4. Literature lacks studies on the influence of Ni on the crystallisation sequence of amorphous AlO*_x_* H*_y_* with or without H. Tkalec et al. [41] applied differential scanning calorimetry (DSC), XRD, and (scanning) transmission electron microscopy (S/TEM) to investigate the role of Ni (0–3 at.%) replacing Al in amorphous diphasic Al_2_O_3_ – SiO_2_ precursors for sol‐gel manufacturing. DSC data showed that addition of 3 at.% Ni facilitated the crystallisation of γ‐Al_2_O_3_ at 874°C, which was not visible for samples with 0, 1, and 2 at.% Ni. Their findings therefore clearly agree with an earlier onset temperature for crystallisation of Ni‐containing Al_2_O_3_. In another study, crystallisation of ∼3 Å amorphous Al_2_O_3_ thin films grown on (100) NiAl substrates was reported to be temperature and time sensitive in the annealing temperature range of 450°C to 500°C [42], significantly lower than common ALD‐Al_2_O_3_ crystallisation temperatures [32]. A well‐established phenomenon that can markedly reduce the thermal stability of crystalline / amorphous laminates is metal‐induced crystallisation (MIC), where facilitated crystallisation of an adjacent amorphous layer can generally be linked to a low‐energy interface with a high density of defects and high diffusivity in the crystalline part [43]. As the Al_100‐z_Ni_z_ / AlO*_x_* H*_y_* nanolaminates offer a large amount of interfaces, Ni‐MIC needs to be considered. The low bilayer period and crystalline Al‐Ni adjacent to an ionic‐covalently bonded amorphous AlO*_x_* H*_y_* layer may indeed lead to bond weakening and higher crystallisation propensity, when the PVD layers are Ni‐doped.

Thus far it appears that the thermal instability of Al_100‐_ *_z_* Ni*_z_* / AlO*_x_* H*_y_* nanolaminates is controlled by the combined effects of room‐temperature ALD (with its pronounced H incorporation) and the presence of Ni in the (crystalline) layers. The detrimental effect of Ni on the thermal stability is of particular interest and in direct contrast to beneficial Ni‐induced strengthening effects in the as‐deposited state [33]. This complex behaviour is examined and elaborated in greater detail in the following chapters.

### 2.2 Phase‐Interface Competition in Al‐Ni

The first limiting factor for thermal stability in Al_100‐_ *_z_* Ni*_z_* / AlO*_x_* H*_y_* thin films is the phase separation of FCC Al_100‐_ *_z_* Ni*_z_* → FCC Al + orthorhombic Al_3_Ni, competing with an interface segregation of Ni solutes in Al. It emphasises the difference between true geometric boundaries (interfaces) and local microstructure and chemistry variations (interphases). The two phenomena are visible in the S/TEM analysis of the evolution of the Al_95_Ni_5_ / AlO*_x_* H*_y_* microstructure after vacuum annealing at *T_hom_* = 0.52 (∼210°C), summarised in Figure 3. Figure 3a shows a representative high angle annular dark field (HAADF)‐STEM image of 24 bilayer periods as well as an selected area electron diffraction (SAED) pattern covering approximately 20 bilayer periods. Automated crystal orientation mapping was performed to resolve the inverse pole figure out‐of‐plane map for FCC Al and a phase map considering FCC Al and orthorhombic Al_3_Ni, visualised in Figure 3b,c. Figure 3d and e further demonstrate the phase separation through high resolution (HR)‐STEM images and selected area fast Fourier transformation (FFT) patterns of an FCC Al and orthorhombic Al_3_Ni grain, respectively. Figure 3f shows an HR‐STEM image focusing on grain boundary segregation including a linescan over the interface, Figure 3g. For chemical mapping, STEM‐energy dispsersive X‐ray spectroscopy (EDS) was performed and Figure 3h–k shows respective HAADF‐, AlO‐, Ni‐yield maps with out‐of‐plane linescan, as well as an in‐plane linescan, respectively.

**FIGURE 3:**
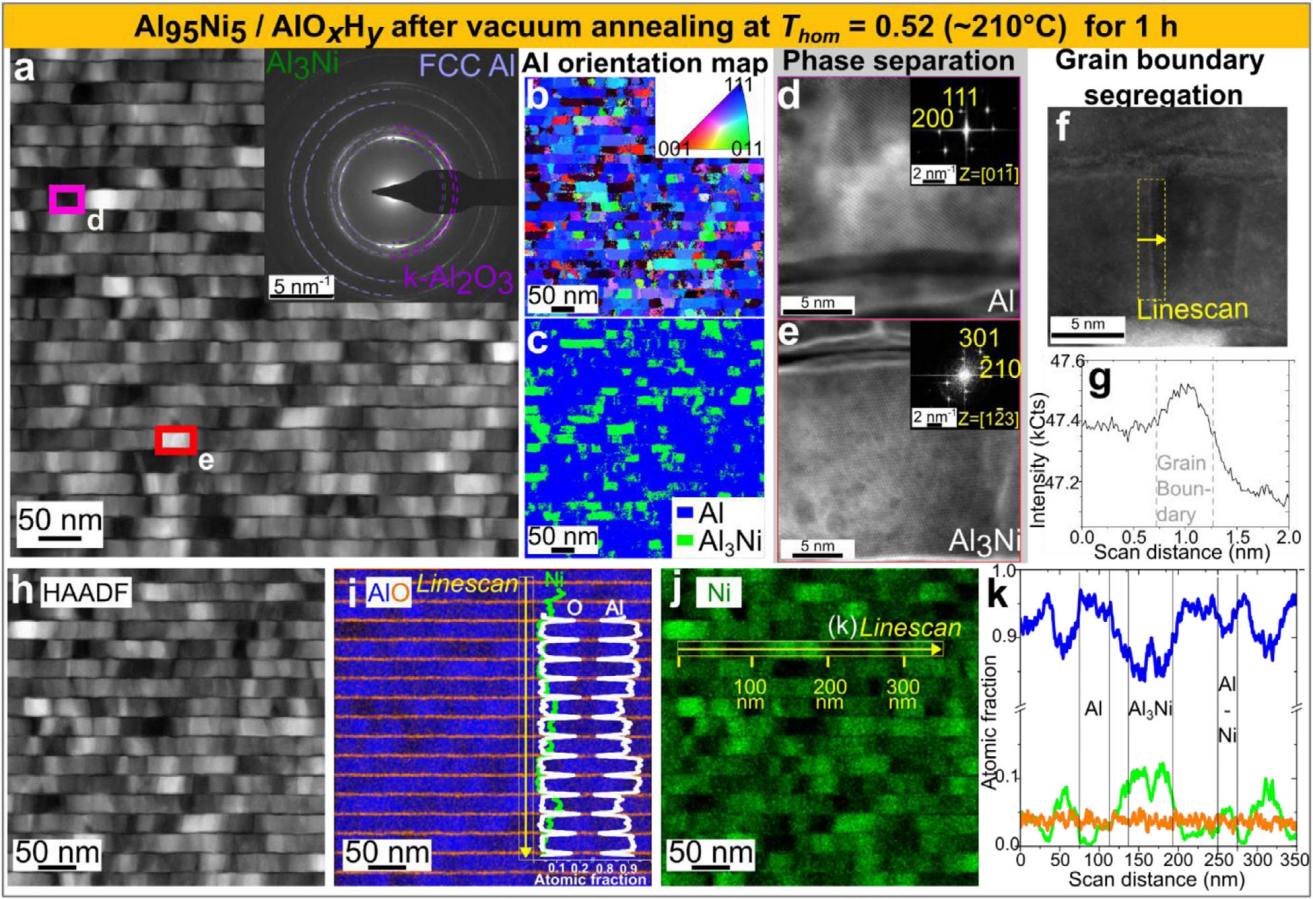
S/TEM results on Al_95_Ni_5_ / AlO*_x_* H*_y_* after annealing at *T_hom_* = 0.52: (a) HAADF‐STEM image of several bilayer periods with SAED pattern, automated crystal orientation mapping (b) inverse pole figure out‐of‐plane map for FCC Al and (c) phase map of Al and Al_3_Ni; HR‐STEM images and selected area FFT patterns of phase separation to (d) FCC Al and (e) orthorhombic Al_3_Ni, (f) HR‐STEM image highlighting grain boundary segregation with (g) linescan; as well as results from STEM‐EDS including (h) HAADF‐yield, (i) Al‐O‐yield and out‐of‐plane linescan, (j) Ni‐yield, and (k) in‐plane line scan. Al appears in blue, Ni in green, and O in orange.

The HAADF‐STEM overview image in Figure 3a confirms the imprint of an intact 25 / 1 nm architecture of crystalline Al‐Ni layers and continuous AlO*_x_* H*_y_* interlayers where both separation and grain boundary segregation are resolvable. The crystalline layers show a pronounced *Z* contrast, which can be attributed to intermetallic phase formation of Al_3_Ni (Figure 3e). The SAED pattern in Figure 3a confirms the strong (111) FCC Al OP texture, which is additionally confirmed by the automated crystal orientation mapping inverse pole figure out‐of‐plane map in Figure 3b. Moreover, single diffraction events from small volumes can also be derived for the Al_3_Ni phase as well as *κ*‐Al_2_O_3_. IP‐Al crystallite size is in good agreement with synchrotron XRD and a representative DF‐TEM image with objective aperture on the (111) and (200) FCC Al diffractions can be found in Section S3. Compared to the as‐deposited condition, the IP FCC Al grains grow around 290% up to 29 ± 1 nm, with an average IP‐Al_3_Ni grain size of 18 ± 13 nm. Despite Al_3_Ni formation, there is an additional hint toward interface segregation at Al–Al grain boundaries as well as the crystalline / amorphous interfaces (Figure 3c,f). As the S/TEM specimens were exclusively prepared by Xe‐ plasma focused ion beam (pFIB), these artefacts in Figure 3e shall be attributed to a higher *Z* contrast that does not originate from Ga‐FIB preparation [44]. The Al‐O map in Figure 3i confirms continuous AlO*_x_* H*_y_* interlayers with a more pronounced surface roughness, while chemical analysis by STEM‐EDS of 15 interlayers in the very same map reveals a chemical composition of Al_78.0±1.2_Ni_4.5±1.3_O_17.5±0.4_. Thus, there is still Ni detected in the vicinity of the interlayers. The Ni map in Figure 3j indicates Ni‐diffusion and agglomeration in the crystalline Al‐Ni layers. Three different crystalline phases depending on the local Ni content are formed, which is confirmed by the in‐plane linescan in Figure 3k: almost Ni‐deficient FCC grains, FCC Al‐Ni solid solution, as well as orthorhombic Al_3_Ni grains. Their chemistries, excluding remaining O, can be quantified as Al_96.4±0.9_Ni_0.5±0.3_, Al_90.7±2.1_Ni_5.5±1.2_, and Al_82.0±1.8_Ni_14.3±0.7_, respectively.

Vacuum annealing at *T_hom_* = 0.52 (∼210°C) was chosen due to previously reported Mg‐enrichment of nanocrystalline Al–Al grain boundaries in Al_100‐_ *_z_* Mg*_z_* (*z* ≤ 7 at.%) when annealed at *T_hom_* = 0.51 (∼200°C) for 1 h and subsequent strength increase [45]. Similarly, Balbus et al. [16] reported amorphous Ni‐Ce formation in a nanocrystalline AlNiCe alloy promoting enhanced thermal stability and mechanical properties. The authors proposed intragranular amorphous phase formation sufficiently hindering the intergranular formation of the intermetallic Al_11_Ce_3_ and Al_3_Ni, while Al_3_Ni was not observed below 300°C. In another study on Al_93.5_Mn_6.5_, Mn segregation at Al–Al grain boundaries was suggested as a reason for stability at *T_hom_* = 0.51 (∼200°C), while stability at *T_hom_* = 0.61 (∼300°C) occurred despite dual‐phase microstructure and Al_6_Mn precipitation [4]. Here, Ni diffusion in Al_95_Ni_5_ / AlO*_x_* H*_y_* nanolaminates seems to indeed trigger interface segregation to both Al grain boundaries and the Al / AlO*_x_* H*_y_* interfaces (Figure 3d). Such interface complexions in doped systems can appear in various ways: clean, monolayer, bilayer, trilayer, nanolayer, or wetted layer; ordered and disordered [10, 30]. A potentially ordered single atomic layer Ni segregation on Al–Al grain boundaries like the one in Figure 3d would need to follow epitaxial growth on the Al plane [46], which makes plane spacing measurements from the STEM image redundant. However, the plot profile of the grain interior to grain boundary area in Figure 3g shows slightly increased intensity for the atomic layer adjacent to the Al grain boundary.

Yet, interface segregation effects here are mostly prevented by the formation of the thermodynamically more stable Al_3_Ni phase. The formation of intermetallic Al_3_Ni might decelerate grain growth and thereby affect the evolution of IP‐Al crystallite size. Figure 1b shows almost constant IP‐Al crystallite size until *T_hom_ =* 0.46 (∼160°C) for Al_95_Ni_5_ / AlO*_x_* H*_y_*, yet a strong increase of IP‐Al crystallite size, when Ni‐doped films were annealed at *T_hom_ =* 0.52 (∼210°C). Here, this strong relative change likely originates from the formation and / or growth of Al_3_Ni upon annealing originating from Ni‐rich nanoclusters in the as‐deposited condition. Huang, Kalidindi, and Schuh [4] reported grain size retention below 100 nm partially due to phase formation of Al_6_Mn in an electrodeposited nanocrystalline Al_93.5_Mn_6.5_ alloy. Here, the Al_3_Ni formation can only be confirmed by SAED at *T_hom_ =* 0.52 (∼210°C) (Figure 3a), but not by XRD at *T_hom_ =* 0.77 (∼450°C) (Figure 2). Yet, the Al_3_Ni phase is anticipated as an Al grain stabiliser in a Zener pinning mechanism only until *T_hom_ =* 0.46 (∼160°C) for 1 h; however upon Al_3_Ni formation and growth, the relative IP‐Al grain growth in Ni‐doped films exceeds the undoped reference film (Figure 1b). Notably, the enthalpy of Al_3_Ni formation is approximately −60 kJ mol^−1^ [35], while the enthalpy of Ni segregation at Al–Al grain boundaries lies in the range from 0 to −25 kJ mol^−1^ [47]. Therefore, under these annealing profiles close to thermodynamic equilibrium, the formation of Al_3_Ni is expected to be more favourable than Ni segregation at Al–Al grain boundaries. However, it should be emphasised that Al grain size in our study at similar annealing temperature and duration (∼200°C for 1 h) lies below 30 nm, which is significantly lower than 100 ± 50 nm for the Al_93.5_Mn_6.5_ alloy [4]. Recently, Cunningham et al. [48] postulated the effect of flash DSC with varying cyclic cooling rates up to 10.000°C s^−1^ successfully enabling amorphous Al‐Ni phases at Al–Al grain boundaries in a sputter‐deposited Al_98_Ni_2_ alloy. Therefore, the effect of such metastable heating profiles on the phase formation in the Al_100‐_ *_z_* Ni*_z_* / AlO*_x_* H*_y_* here remains an open question.

### 2.3 Nanoscale Chemistry

We have previously reported the enrichment of S/TEM(‐EDS) datasets through APT on the as‐deposited films that elucidated nanoscale composition data not‐resolvable by S/TEM [33]. The screening by XRD (Figure 2) has already marked the crystallisation of AlO*_x_* H*_y_*. Here, APT offers the unique ability to track the evolution of matrix and alloying elements, including O and even H in 3D for correlation to the stability of the interlayers. Therefore, a cross‐correlation of S/TEM and APT on the role of Al, Ni, O, and H is conducted in the following. The Al_95_Ni_5_ / AlO*_x_* H*_y_* at *T_hom_ =* 0.32, 0.52, and 0.77 (as‐deposited with ∼23°C as well as annealed at ∼210°C and ∼450°C) were chosen as films of particular interest. Figure 4 shows reconstructions of Al, Ni, O, and H atomic positions of two bilayer periods in each condition and respective OP composition profiles from cylinders over two Al_95_Ni_5_ / AlO*_x_* H*_y_* interfaces. Isoconcentration surfaces of 10, 5, and 10 at.% for Ni, O, and H, respectively, were chosen to visualise local enrichment of the respective elements. It should be noted that the H content can be understood as an upper boundary value due to the likely contribution of residual gas from the analysis chamber. The reconstructions correspond to slices of 10 nm and enable illustration on the order of one single grain depth. Similar phenomena were observed for Al_98_Ni_2_ / AlO*_x_* H*_y_* at *T_hom_ =* 0.52 (∼210°C) and corresponding data can be found in Section S4. Mass‐spectra for Al_95_Ni_5_ / AlO*_x_* H*_y_* are displayed in Section S5.

**FIGURE 4:**
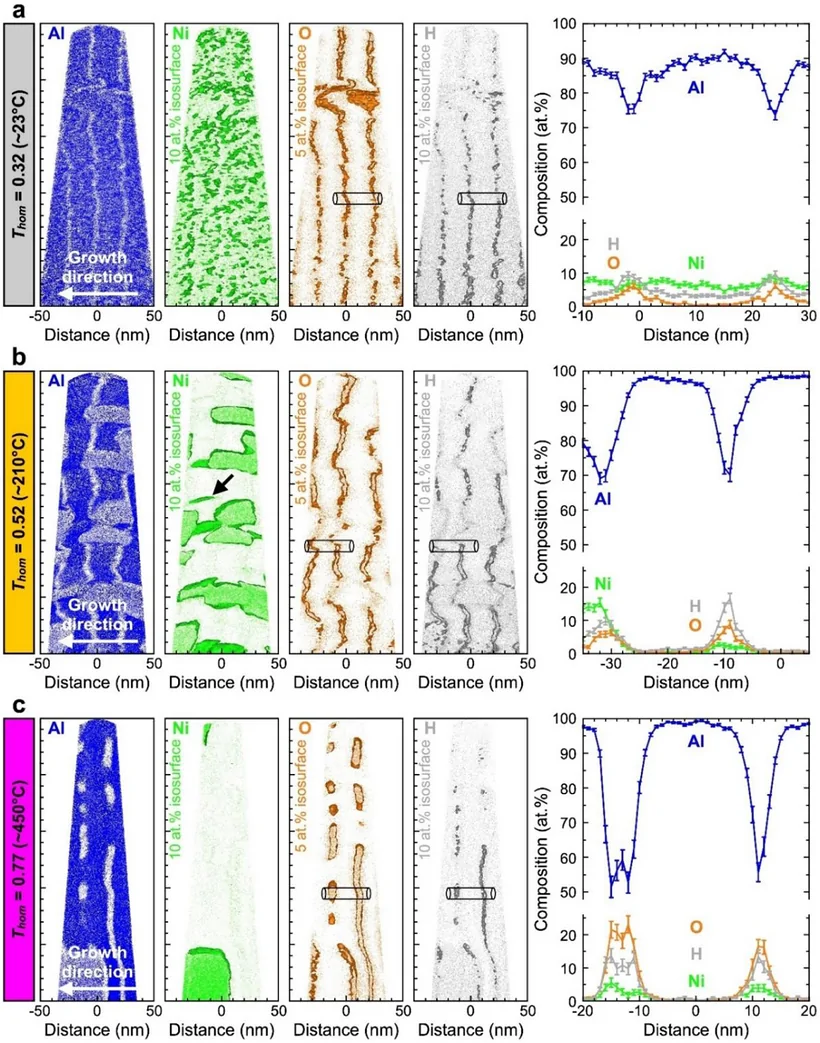
APT reconstructions of atomic positions (10 nm slices) including Al, Ni, O, and H as well as composition profiles for Al_95_Ni_5_ / AlO*_x_* H*_y_* at three selected annealing conditions: (a) *T_hom_* = 0.32 (as‐deposited, adapted from [33]), (b) *T_hom_* = 0.52 (∼210°C), and (d) *T_hom_* = 0.77 (∼450°C); as well as respective out‐of‐plane composition profiles from cylinders. Al appears in blue, Ni in green, O in orange, and H in gray. The black arrow marks most likely a Ni‐rich grain boundary.

In the as‐deposited condition, the crystalline Al‐Ni layers consist of a Ni‐deficient solid solution and randomly distributed Ni‐rich nanoclusters. The adjacent AlO*_x_* H*_y_* interlayers show coinciding O and H signals that build up quasi‐continuous interlayers. For further details readers are referred to our previous publication [33]. At *T_hom_* = 0.52 (∼210°C), the crystalline layers show Ni‐rich volumes, which are consistent with individual grains and seem to impinge and bow the AlO*_x_* H*_y_* interlayers. Contiguous AlO*_x_* H*_y_* interlayers exhibit reduced continuity, a certain broadening as well as differences in O‐ and H‐enrichment. Additionally, there is a certain decoupling observable between composition peaks of O and H, when the AlO*_x_* H*_y_* interlayers collide with the Ni agglomeration zones, indicating reduced AlO*_x_* H*_y_* stability in the vicinity of Ni‐enriched volumes. Notably, the 10 at.% Ni isosurface for *T_hom_* = 0.52 in Figure 4c shows one Ni‐enriched interface (annotated with a black arrow), which is in good agreement with the more pronounced *Z* contrast at a vertical Al–Al grain boundary visualised through HR‐STEM in Figure 3d. The measurement cylinder reveals a quasi‐similar chemical composition of that Ni‐enriched interface and the Al_3_Ni phases. Therefore, and in contrast to the analysis of as‐deposited films, STEM‐EDS and APT show good agreement on the competing mechanism of Ni segregation and the phase separation to Al and Al_3_Ni upon thermal annealing. Lei et al. [11] reported the formation of amorphous grain boundary complexions and amorphous core‐shell nanorods in ball‐milled nanocrystalline Al‐Mg‐Y, Al‐Fe‐Y, and Al‐Ni‐Y. Notably, the core–shell nanorods were found to nucleate at grain boundaries. Likewise, we are assuming Ni diffusion from Ni‐rich nanoclusters toward Al‐Al grain boundaries, which results in the observed enrichment. Figure 3f however, demonstrates that the atomic arrangement of the Ni‐segregation at the Al–Al grain boundary is not confirmed to have an amorphous nature and neither several nanometres of thickness. Then, with sufficient Ni gathered, the Al_3_Ni phase can precipitate and grow from the grain boundary into the adjacent matrix.

Upon further annealing at *T_hom_* = 0.77 (∼450°C), both crystalline Al_100‐_ *_z_* Ni*_z_* and amorphous AlO*_x_* H*_y_* layers alter significantly. In the crystalline layer, the Ni‐deficient Al matrix is growing in both OP and IP directions, and a larger Ni‐rich volume is visible. The Ni‐enrichment appears to be still confined by neighbouring AlO*_x_* H*_y_*, although it distorts the Al grain underneath, but no Ni‐segregation at vertical Al‐Al grain boundaries is visible anymore. One important aspect of PVD / ALD nanolaminating is the stabilisation of PVD‐derived microstructure toward enhanced thermal stability. Multilayered Al / Ni systems without e.g., AlO*_x_* H*_y_* interlayers are commonly subject to chemical mixing at the interfaces and formation of Al‐Ni compounds (upon annealing) such as the primary formation of Al_3_Ni at ∼270°C [49]. The authors emphasised the role of triple junctions serving as heterogeneous nucleation sites, where upon annealing, Al_3_Ni can grow and coalesce as a continuous layer to reduce the thermal stability of Al / Ni films. Such coalescent Al_3_Ni formation can be prevented here by ALD interlayers, which is a sign toward more effective chemical control by thermally exposed PVD / ALD films.

At *T_hom_* = 0.77 the AlO*_x_* H*_y_* interlayer broadening continues, and interlayer breaking is visible with several tens of nanometres spacing between AlO*_x_* H*_y_* segments. The remaining AlO*_x_* H*_y_* segments additionally show a more pronounced decoupling of O and H signal in a sort of core‐shell structure. Despite an effective spatial resolution limit of 1 nm by APT [50], the relative changes of AlO*_x_* H*_y_* annealing indicate a volume expansion in correlation with instability, which will be subject of the discussion in Section 2.4.

The chemistries of Al grains can be derived to approximately Al_87.9±2.6_Ni_6.8±1.5_O_1.2±0.6_H_4.1±1.1_, Al_97.2±0.6_Ni_0.8±0.3_O_0.4±0.1_H_1.6±0.3_, and Al_100_ for *T_hom_* = 0.32, 0.52, and 0.77, respectively. Therefore, Al grains continuously become Ni, O, and H‐deficient upon annealing. The Ni‐rich volumes can be quantified to Al_77.1±1.3_Ni_20.9±1.2_O_0.2±0.2_H_1.8±0.5_ at *T_hom_* = 0.52 and Al_70.0±1.8_Ni_25.0±2.1_O_1.0±0.4_H_4.0±1.3_ at *T_hom_* = 0.77. Thus, the stoichiometry of Al_3_Ni with O and H impurities is reached upon annealing. However, upon annealing, the H content in the Ni‐rich grains is continuously increasing, while it is decreasing for the Al grains. The cross‐correlation of STEM and APT link the Ni‐rich grains to intermetallic Al_3_Ni, which is in agreement with recent studies of intermetallic alloys as hydrogen getters [51], although Al_3_Ni was not‐yet‐reported as a hydrogen‐getter material and no hydride formation is observed here through diffraction.

In contrast, the AlO*_x_* H*_y_* concentrations evolve in the following way from *T_hom_* = 0.32 to 0.52 and 0.77: Al_75.0±0.7_Ni_8.5±1.1_O_5.9±0.8_H_10.6±1.6_, Al_70.2±2.9_Ni_4.0±1.2_O_10.8±1.2_H_15.0±2.1_, and Al_62.1±3.9_Ni_4.1±1.2_O_21.3±2.6_H_12.5±1.7_. Upon annealing, the interlayers are continuously detected to be O‐deficient, but progression toward the ideal Al_2_O_3_ chemistry with H‐contamination can be observed.

Beyond the enhanced structural integrity, Al / *α*‐Al_2_O_3_ coatings were recently proposed as promising candidates for H barrier coatings [52]. Therefore, the role of H for the stability of the Al_100‐_ *_z_* Ni*_z_* / AlO*_x_* H*_y_* architectures is of particular interest. Here, the bulk compositions, considering the entire APT specimens (Figure 4), reveal H outdiffusion from the Al_95_Ni_5_ / AlO*_x_* H*_y_* specimens: average (upper boundary) H concentrations decrease from 4.2 at.% to 2.7 at.% to 1.2 at.% for *T_hom_* = 0.32, 0.52, and 0.77, respectively. Figure 4b,d, and f give an overview of the Al, Ni, O and H compositions over two interfaces in Al_95_Ni_5_ / AlO*_x_* H*_y_* derived from the APT reconstructions in Figure 4 and measurement cylinders with 10 nm diameter. The composition profiles reveal strong composition variations of both Al_100‐_ *_z_* Ni*_z_* (Al + Al_3_Ni) and AlO*_x_* H*_y_* upon annealing. Here, focus is shed on the analysis of light elements O and H, which are decreasing in Al_100‐_ *_z_* Ni*_z_* upon annealing. Upon partial crystallisation and breaking at *T_hom_* = 0.77 (∼450°C), the Al_100‐_ *_z_* Ni*_z_* / AlO*_x_* H*_y_* interface additionally becomes sharper. Al / α‐Al_2_O_3_ coatings by (reactive) magnetron sputtering were proposed as H permeation barriers at ambient temperature due to enhanced damage resistance and low H diffusivity [52]. Complementary density‐functional theory (DFT) and Grand Canonical Monte Carlo simulations unravelled the root cause in superabundant (several orders of magnitude increased [53]) metal Al vacancy concentrations at crystalline / crystalline Al / α‐Al_2_O_3_ interfaces that allow H trapping, while H barrier performance scales with increasing amount of Al / Al_2_O_3_ interfaces. The roughly 114 Al_100‐_ *_z_* Ni*_z_* / AlO*_x_* H*_y_* interfaces through the ∼ 3 µm 25 / 1 nm bilayer period might indeed induce similar H permeation barrier capabilities, but the AlO*_x_* H*_y_* interlayers are already H‐enriched and DFT simulations predict slightly accelerated diffusivity of H in amorphous AlO*_x_* H*_y_* [54]. H diffusivity in Al at room‐temperature can be estimated to 1.5×10^−14^ m^2^ s^−1^ [55], which is several magnitudes higher than H diffusion in any AlO*_x_* H*_y_* [54] and explains the apparent H diffusion visualised through APT in Figure 4b for Al_95_Ni_5_ / AlO*_x_* H*_y_* annealed at *T_hom_* = 0.52 (∼210°C). We are expecting here further accelerated room temperature diffusivity in the AlO*_x_* H*_y_* layers deposited by room‐temperature ALD, due to a significantly lower density of < 2.4 g cm^−3^ compared to 3.2 g cm^−3^ for close‐to stoichiometric Al_2_O_3_ [40], which creates more diffusion paths. Thus, amorphous ALD‐AlO*_x_* H*_y_* is seen as less promising candidate for multi‐layered H barrier coatings. The question remains on the H barrier performance of post‐annealed Al_100‐_ *_z_* Ni*_z_* / AlO*_x_* H*_y_*, which are subject to AlO*_x_* H*_y_* decomposition and will be more discussed in Section 2.4.

On the cross‐correlation of XRD, S/TEM, and APT at *T_hom_* = 0.52, there are several points to be remarked. Generally, both S/TEM and APT agree on the formation of Ni‐rich volumes. Here, S/TEM and XRD confirmed Al_3_Ni crystallinity and APT clarified the impinging character on the adjacent AlO*_x_* H*_y_* interlayers, which is especially visible at *T_hom_* = 0.52 in Figure 4b. S/TEM in Figure 3 indicates slightly enhanced roughness of the AlO*_x_* H*_y_* interlayers after annealing compared to the as‐deposited state, where APT hints toward increased surface roughness. Notably, this is not seen as an indicator for breaking of the interlayers at *T_hom_* = 0.52 due to continuous O‐ and H enrichment, though with more pronounced chemical heterogeneity (less continuous excess as revealed by isoconcentration surfaces) compared to the as‐deposited condition. The results from XRD in Figure 2 have shown the formation of superlattice fringes around the (111) FCC Al peak at 2*θ* of 38.6° after annealing at *T_hom_ ≥* 0.61 (300°C) for 1 h only in 5 at.% Ni doped films. Although superlattice fringes are usually seen as sign for excellent structural coherency and smooth interfaces in multi‐layered or epitaxial thin films [56, 57], the superlattice fringes here only appear upon AlO*_x_* H*_y_* roughening. Hence, the superlattice fringes are assumed to stem from the formation of a thin adjacent FCC Ni layer from Ni outdiffusion, which is in good agreement with the higher *Z* contrast layers in HAADF‐STEM images in Figures 3 and 5 (presented in the next Section 2.4).

**FIGURE 5:**
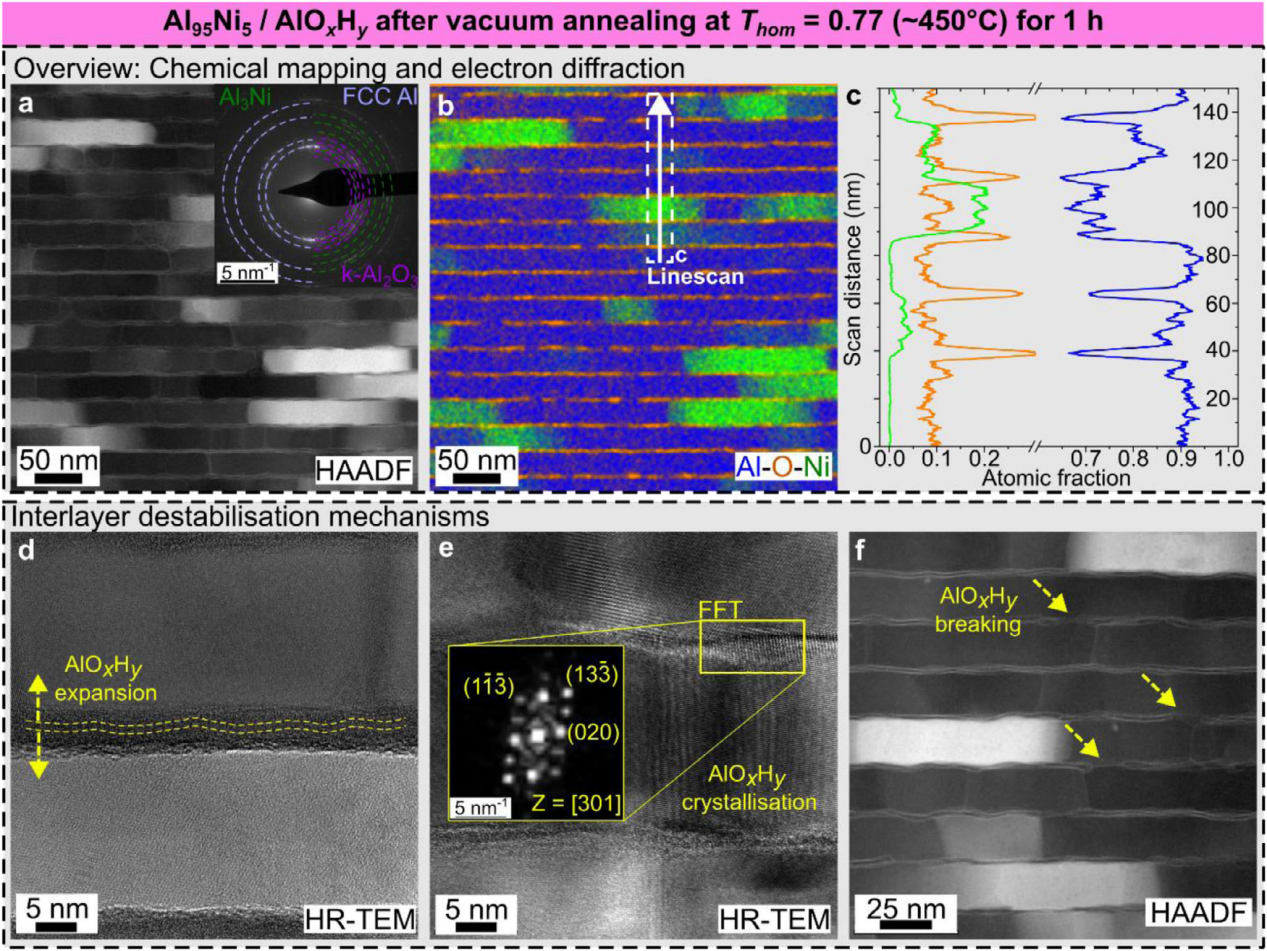
S/TEM results on Al_95_Ni_5_ / AlO*_x_* H*_y_* after annealing at *T_hom_* = 0.77 (∼450°C): S/TEM overview (a) HAADF‐yield with SAED pattern, (b) Al‐O‐Ni‐yield, and (c) corresponding out‐of‐plane EDS line scan, as well as overview of interlayer destabilisation mechanisms (d) expansion, (e) partial crystallisation toward *κ*‐Al_2_O_3_, and (f) breaking points.

After elucidating the role of intermetallic Al_3_Ni phase formation and roughening of the AlO*_x_* H*_y_* interlayers on the microstructure stability and IP‐Al crystallite size, further assessment is needed to unravel the effect and mechanism of AlO*_x_* H*_y_* interlayer breaking and its implications for OP‐grain growth.

### 2.4 Interlayer Breaking and Dewetting

Significant AlO*_x_* H*_y_* interlayer breaking tendencies in Al_95_Ni_5_ / AlO*_x_* H*_y_* at *T_hom_* = 0.77 were resolved by APT in Figure 4c. The previous discussion has unraveled increased destabilisation tendencies through H‐enrichment in the AlO*_x_* H*_y_* and Ni diffusion. Therefore, cross‐sectional S/TEM analysis was performed for both Al_98_Ni_2_ / AlO*_x_* H*_y_* and Al_95_Ni_5_ / AlO*_x_* H*_y_* after annealing at *T_hom_* = 0.77 for 1 h. Figure 5 illustrates an overview of STEM(‐EDS) results for Al_95_Ni_5_ / AlO*_x_* H*_y_* (Figure 5a–c) and respective interlayer breaking mechanisms (Figure 5d–f). Representative STEM‐EDS images for Al_98_Ni_2_ / AlO*_x_* H*_y_* after annealing at *T_hom_* = 0.77 for 1 h are displayed in Section S6 and emphasise more continuous and therefore stable AlO*_x_* H*_y_* layers.

The HAADF‐STEM image of several bilayer periods Al_95_Ni_5_ / AlO*_x_* H*_y_* after vacuum annealing *T_hom_* = 0.77 (∼450°C) for 1 h in Figure 5a confirms the ongoing phase separation between crystalline FCC Al and orthorhombic Al_3_Ni as well as the in‐plane grain growth of both phases. Contrary to three crystalline domains in the Al‐Ni layers at *T_hom_* = 0.52 (∼210°C, Figure 3k), the OP EDS linescan in Figure 5c confirms two remaining domains: almost pure Al grains and Al_3_Ni grains. Their chemistries are quantified as Al_90.4±2.3_Ni_0.2±0.2_O_9.4±1.8_ and Al_71.6±3.4_Ni_18.1±1.8_O_10.3±2.4_, similar to pure Al and Al_3_Ni with O impurities. Additionally, SAED in Figure 5a confirms the crystalline nature of *κ*‐Al_2_O_3_. However, this crystallisation is seen here as partial crystallisation, since there is no evidence that all AlO*_x_* H*_y_* layers have crystallised. The *Z* contrast among interfaces is more pronounced, and the interlayers are broader and less continuous than at lower annealing temperatures. Notably, the Al‐O‐Ni maps in Figure 5b annotate interlayer breaking due to O‐yield discontinuity. Additionally, the out‐of‐plane EDS linescan over a couple of Al_95_Ni_5_ / AlO*_x_* H*_y_* bilayer periods in Figure 5c confirms the lack of O‐enrichment and thus, interlayer discontinuity with the formation of interlayer segments. These discontinuities are in good agreement with APT results in Figure 4, which provided evidence for destabilisation of Al_95_Ni_5_ / AlO*_x_* H*_y_* at *T_hom_* = 0.77 (450°C). The more pronounced destabilisation of amorphous AlO*_x_* H*_y_* interlayers for samples doped with 5 at.% Ni compared to 0 and 2 at.% Ni is consistent with the results from (synchrotron) XRD that showed more pronounced Al_2_O_3_ crystallinity compared to the undoped sample.

Following the results in Figure 5d–f, we can determine three AlO*_x_* H*_y_* interlayer degradation mechanisms on the example of Al_95_Ni_5_ / AlO*_x_* H*_y_* at *T_hom_* = 0.77: volume expansion, crystallisation, and breaking. Bright field (BF)‐TEM images in Figure 5d,e confirm AlO*_x_* H*_y_* volume expansion (manifested as a layer‐thickening) up to 2.3 ± 0.5 nm and partial crystallisation toward *κ*‐Al_2_O_3_ confirmed by selected area FFT in the interlayer vicinity. It is noted that the BF‐TEM image in Figure 5d illustrates an example of extraordinary layer swelling up to ∼4.7 nm. Additionally, Figure 5f shows discontinuous AlO*_x_* H*_y_* interlayers (indicated by yellow arrows) that give rise to the formation of Al–Al grain boundaries and facilitated grain growth in the out‐of‐plane direction. The here observed degradation of 1 nm amorphous AlO*_x_* H*_y_* layers, fabricated by ALD, disagrees with commonly observed crystallisation of ALD‐AlO*_x_* H*_y_* only above 750°C, depending on annealing time, film thickness and substrate, respectively [32], as already mentioned in the discussion of XRD results in Section 2.1. Local crystallisation of thin amorphous specimens can be a consequence of electron irradiation during TEM at 200 kV [34]. Yet, the results from (synchrotron) XRD allow a determination on *κ*‐Al_2_O_3_ crystallisation temperature at *T_hom_* > 0.61 (∼300°C).

Hence, the remaining question needs to focus on the sequence of expansion – crystallisation – breaking of the AlO*_x_* H*_y_* interlayers. It is emphasised that volume expansion (Figure 5d) and formation of crystallisation sites (Figure 5d) in AlO*_x_* H*_y_* upon annealing are mechanisms originating from room temperature ALD and H enrichment [39, 58, 59]. Such solid AlO*_x_* H*_y_* complexes can indeed contain up to 65%–75% of water [60], which is confirmed by the low density of room‐temperature AlO*_x_* H*_y_* < 2.4 g cm^−3^ compared to stoichiometric Al_2_O_3_ (3.2 g cm^−3^) [39] and water (1.0 g cm^−3^). It is remarked that hydrogen‐containing species incorporated during room‐temperature ALD—whether as molecular H_2_O, hydroxyl groups, or other H‐related defects—are difficult to distinguish experimentally, especially in 3D and ex situ [61]. Additionally, while the incorporated hydrogen originates partially from the H_2_O precursor during room‐temperature ALD [39], its exact chemical state after deposition is uncertain here. Consequently, the following discussion addresses the collective thermal mobilisation of hydrogen‐containing species during annealing and contribution to the observed microstructural evolution solely as *hydrogen‐mediated* mechanisms of *hydroxylated species*. Therefore, quartz crystal microbalance (QCM) measurements were conducted for ALD‐AlO*_x_* H*_y_* at room temperature by trimethylaluminium (TMA) and deionised (DI) scanning ‐H_2_O. Mass gains for three ALD cycles are displayed in Section S7. The results indicate a mass gain of 9.3 ± 0.2 ng cm^−2^ upon the pulse of DI‐H_2_O at room temperature, an indicator for the physisorption subsequent hydroxylation. Henceforth, hydrogen‐mediated volume expansion (swelling) upon annealing at low temperatures is suggested by the combined QCM, APT, and S/TEM observations that indicate the initiation of AlO*_x_* H*_y_* decomposition. Sato [62] accordingly reported the thermal decomposition temperature of amorphous AlO*_x_* H*_y_* precipitates from aqueous solutions, more specifically amorphous and pseudo‐boehmite AlO(OH), by differential thermal analysis around 250–300°C, followed by the subsequent formation of amorphous Al_2_O_3_. Decomposition of AlO*_x_* H*_y_* then gives rise to various possible scenarios, two of the main ones include the reaction of *hydroxylated species* with adjacent phases or evaporation from the amorphous layers. Notably, hydrolysis can be assumed due to quasi‐linear AlO*_x_* H*_y_* thickness increase from 1.1 ± 0.2 to 1.4 ± 0.3, and 2.3 ± 0.5 nm for Al_95_Ni_5_ / AlO*_x_* H*_y_* annealed at *T_hom_* = 0.32, 0.52, and 0.77, respectively. However, the formation of bubbles on the surface of the films after annealing is observed, which is an indicator for evaporation of hydroxylated species and in good agreement with decreasing O and H contents in APT specimens upon annealing. Representative images from scanning electron microscopy (SEM) investigation are displayed in Section S8. Then, crystallisation of AlO*_x_* H*_y_* is a compression‐induced phase decomposition at heterogeneous nucleation sites from a less‐dense to more‐dense structure with an imminent molar volume change [39, 59], and thus needs to result in volume contraction. Ultimately, this crystallisation is estimated to result in stress release and interlayer breaking [63], giving rise to out‐of‐plane grain growth as grain boundaries replace the crystalline / amorphous interfaces.

Here, ex situ analysis of residual stress in the metallic components of the nanolaminates (after cooling and therefore stress relaxations) was performed using the sin^2^ψ method. The error bars in residual stress are derived from the R‐squared value of the fitted linear trendline of the ln(1/sin(*θ*)) over sin^2^ψ dataset for each condition.

The results in Figure 6 shows increasing tensile residual stress (*σ_res_*) with increasing vacuum annealing temperature for all three Al_100‐_ *_z_* Ni*_z_* / AlO*_x_* H*_y_* thin film architectures. Only the as‐deposited Al_95_Ni_5_ / AlO*_x_* H*_y_* has a slightly compressive residual stress of *σ_res_* = ‐82 ± 60 MPa. For the Al / AlO*_x_* H*_y_* films, the residual stress is linearly increasing from *T_hom_* = 0.32 to 0.94 and *σ_res_* = 125 ± 22 MPa to 540 ± 2 MPa, respectively. For Ni‐doped films, the residual stress is almost linearly increasing only until *T_hom_* = 0.77 with residual stress values for Al_98_Ni_2_ / AlO*_x_* H*_y_* and Al_95_Ni_5_ / AlO*_x_* H*_y_* ranging from *σ_res_* = 56 ± 42 to 563 ± 6 MPa and *σ_res_* = ‐82 ± 60 to 532 ± 5 MPa, respectively. In contrast to pure Al layers, further annealing at *T_hom_* = 0.94 for 1 h induces residual stress reduction for 2 and 5 at.% Ni doped films toward *σ_res_* = 416 ± 36 and 395 ± 7 MPa, respectively.

**FIGURE 6:**
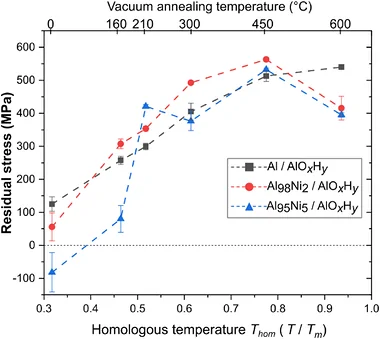
Ex situ residual stress of the Al_100‐_ *_z_* Ni*_z_* / AlO*_x_* H*_y_* thin films as a function of homologous temperature / vacuum annealing temperature for 1 h derived from sin^2^ψ XRD.

Due to differences in thermal expansion between individual constituents of the Al_100‐_ *_z_* Ni*_z_* / AlO*_x_* H*_y_* thin films and the Si substrate, thermal stress cannot be neglected. The evolution of thermal stress within the nanolaminates can be quantified by using the following simplified relationship:

(1) $$\sigma_{thermal}=\frac{E_{film}}{1-\nu_{film}}\left(\alpha_{si}-\alpha_{film}\right)\left(T-T_{d}\right)$$

with thermal stress *σ_thermal_*, Young's modulus *E_film_*, Poisson's ratio *ν_film_*, Si substrate thermal expansion coefficient (CTE) *α_Si_* (2.55×10^−6^–4.10×10^−5^ K^−1^), film CTE *α_film_* (2.65×10^−5^–3.55×10^−5^ K^−1^), and temperature difference from deposition temperature *T_d_* (23°C) and vacuum annealing temperature *T* (160°C to 600°C) to room temperature. Due to the marginal volume fraction of AlO*_x_* H*_y_*, *E_film_*, *ν_film_*, and *α_film_* are assumed as *E_Al_*, *ν_Al_*, and *α_Al_* from an Al film. Henceforth derived thermal stress from the cooling down after annealing to 160°C, 210°C, 300°C, 450°C, and 600°C are all of compressive nature and calculated to 328 MPa< *σ_thermal_* ≤ 430 MPa, 448 MPa< *σ_thermal_* ≤ 587 MPa, 663 MPa< *σ_thermal_* ≤ 870 MPa, 1023 MPa< *σ_thermal_* ≤ 1341 MPa, and 1382 MPa< *σ_thermal_* ≤ 1812 MPa, respectively. Calculated thermal stresses exceed the residual stresses for every annealing condition. Notably, stress relaxation and recovery upon cooling will result in lower residual stress obtained from ex situ sin^2^ *ψ* than calculated thermal stress. However, the order of stress magnitude for both residual and thermal stress is expected to give rise to plastic deformation in the Al_100‐_ *_z_* Ni*_z_* layers. In fact, the phase‐separated Al and Al_3_Ni grains in this particular grain size regime are expected to possess yield stress in the range of several hundred MPa [64]. The assumed plastic deformation in the crystalline Al and Al_3_Ni phases upon annealing at temperatures higher than 450°C will therefore contribute to the limited thermal stability of the architectures probed here. It is reasonable to expect that the tensile stress will additionally facilitate diffusion and increase grain migration tendencies [65], which is supported by the abnormal grain growth at *T_hom_* > 0.77 (see Figure 1).

Ultimately, the observed crystallisation of 1 nm AlO*_x_* H*_y_* at *T_hom_* > 0.61 (∼300°C) disagrees with crystallisation kinetics expected from literature for ultra‐thin ALD films. Conjunction of intrinsic stress, Ni and H impurities and the interlayer roughness of PVD‐deposited Al_100‐_ *_z_* Ni*_z_* grains allows stress localisation and breaking points. Upon crystallisation from 1 nm amorphous AlO*_x_* H*_y_* to *κ*‐Al_2_O_3_, mechanical stresses in the range of several hundred MPa are expected from the CTEs of Al and Al_2_O_3_ mentioned above.

To probe harsher conditions and microstructure evolution beyond on‐set destabilisation in the range of 300 to 450°C, the samples were also annealed at *T_hom_* = 0.94 (∼600°C). The evolution of Al grain size in Figure 1 gave rise to the strongest grain growth from *T_hom_* = 0.77 to 0.94 both in the OP‐ and IP‐orientation. Due to the destabilising effects of Ni already visible at *T_hom_* = 0.77, the reference Al / AlO*_x_* H*_y_* film was chosen to be investigated more closely by S/TEM. Figure 7 gives an overview of the structure (Figure 7a–c,f) and chemistry (Figure 7d,e) of Al / AlO*_x_* H*_y_* after vacuum annealing at *T_hom_* = 0.94 for 1 h.

**FIGURE 7:**
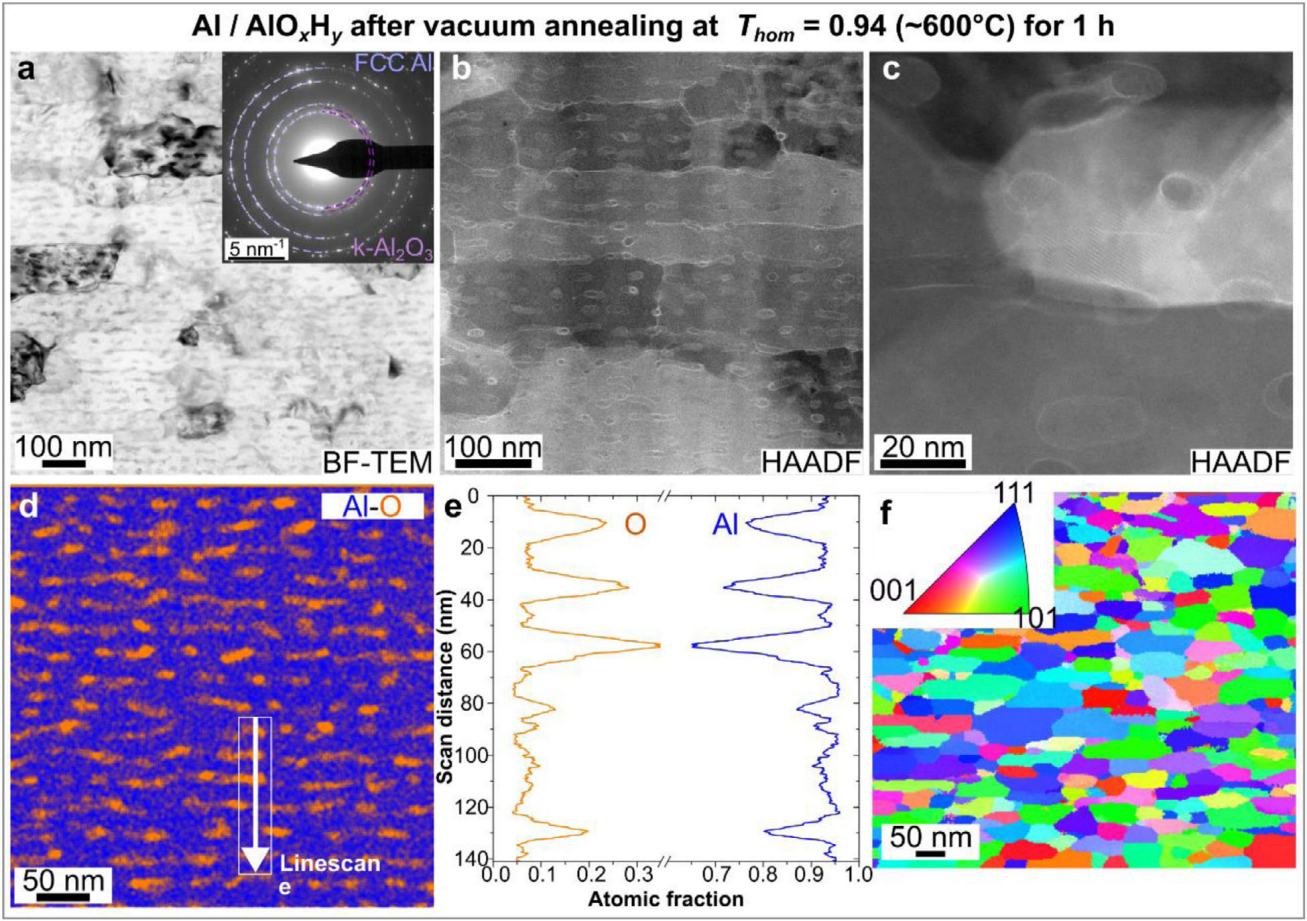
S/TEM results of Al / AlO*_x_* H*_y_* after annealing at *T_hom_* = 0.94 (∼600°C): (a) BF‐TEM image over several original bilayer periods, (b) HAADF‐STEM of several hundred nanometres with SAED inset, (c) higher magnification HAADF‐STEM image; EDS investigation including (d) Al–O yield, and (e) representative out‐of‐plane linescan over a couple of bilayer periods; and (f) automated crystal orientation mapping of inverse pole figure out‐of‐plane map.

The BF‐TEM overview image in Figure 7a shows discontinuous AlO*_x_* H*_y_* interlayers and strong Al grain growth in both out‐of‐plane and in‐plane orientation. The corresponding SAED pattern confirms both FCC Al and orthorhombic *κ*‐Al_2_O_3_ crystallinity. Again, the crystallisation cannot be confirmed for all the Al_2_O_3_ inclusions and is therefore seen as partial crystallisation only. The low amount of Al diffraction events compared to lower annealing temperatures confirms the presence of larger Al crystal sizes of 62 ± 30 nm (derived from crystal orientation mapping of inverse pole figure out‐of‐plane map in Figure 7f). Increasing the magnification, HAADF‐STEM in Figure 7b further confirms the formation of Al‐Al grain boundaries between adjacent sublayers, partially replacing the original AlO*_x_* H*_y_* interlayers. The breaking of AlO*_x_* H*_y_* interlayers gives rise to the encapsulation of mainly spherical AlO*_x_* H*_y_* fragments with an average diameter of 18 ± 7 nm. Note that this size was measured parallel to the long axis for a minimum of 75 AlO*_x_* H*_y_* fragments. The O‐rich fragments are encapsulated within the surrounding Al grains, while some are also located at Al‐Al grain boundaries. Chemically, O‐rich particles and Al‐rich matrix are quantified by STEM‐EDS to Al_71.5±4.2_O_28.5±6.0_ and Al_93.8±1.8_O_6.2±1.3_, respectively. The automated crystal orientation mapping in Figure 7f confirms the broader grain size distribution and equiaxed Al matrix grain structure.

Literature proposes mainly three mechanisms for the thermal instability of crystalline / amorphous nanocomposites, namely MIC [43] as already discussed in Section 2.1, as well as grain boundary grooving [66] / Rayleigh instability [67] in combination with solid‐state dewetting [68]. Thermal grooving commonly requires a polycrystalline nature [66] and as‐deposited AlO*_x_* H*_y_* is amorphous. However, the decomposition and subsequent densification and crystallisation upon vacuum annealing give rise to nanocrystalline domains (Figure 5e) that are expected to allow subsequent grooving. Through the process of dewetting, metastable crystalline thin films frequently evolve into discrete nanoparticles on the substrate surface, driven by void formation and the minimisation of the total free energy [68]. Although dewetting is not expected for amorphous AlO*_x_* H*_y_* due to its still strong bonding character and low mobility, Freddi et al. [69] recently reported on the crystalline nature of Ge islands dewetted from monolithic amorphous Ge films. In the Al / Al_2_O_3_ system, Hieke, Dehm and Scheu [70] reported on the formation of faceted voids through dewetting of crystalline Al with a native amorphous oxide grown on α‐Al_2_O_3_. At 600°C annealing in Ar atmosphere, the crystalline Al film was retracting under the continuous surface oxide to give rise to void formation and pinning of the retracting Al film induced irregular void shapes. Simultaneously the surface oxide was transformed to crystalline γ‐Al_2_O_3_. This observation is similar to the observed microstructure change in phase‐transformed Al_2_O_3_ here, where the spherical shape of Al_2_O_3_ can be a result of competing Al and O mass flux upon annealing. It is worth noting that such dewetted microstructures could still be used to tailor functional material properties. Recently, advances were made by alternating dewetted Au particles with ALD‐Al_2_O_3_ toward disordered metasurfaces for enhanced optical properties [71]. Hence, it remains an open question whether dewetted AlO*_x_* H*_y_* in an architecture like the crystalline / amorphous here could indeed be used for similar applications.

One mechanism not yet considered for the nanolaminated films are phase transformations upon annealing, which release stress. Krautheim et al. [72] investigated mechanical stress in ALD‐Al_2_O_3_ films on Si–SiO_2_/Si_3_N_4_ substrates through wafer curvature measurements during thermal cycling until 870°C and complementary X‐ray techniques. Films with layer thicknesses in the range of 25–60 nm showed a sharp tensile stress increase at 780°C–790°C on the order of 1 GPa, which was linked to volume reduction from crystallisation. Yet, 6 nm films showed a continuous reversible tensile stress rise upon annealing up to almost 6 GPa with no crystallisation detected. Taking the above‐mentioned points into consideration, the thermal instability mechanisms in Al_100‐_ *_z_* Ni*_z_* / AlO*_x_* H*_y_* can be summarised as depicted in Figure 8. Here, Ni exhibits a dual role in the nanolaminates: it enhances as‐deposited hardness but reduces thermal stability by promoting Al_3_Ni precipitation and AlO*_x_* H*_y_* destabilisation. Under the present processing conditions, Ni is therefore not an effective stabilising dopant unless these phase‐separation and oxide‐crystallisation pathways are suppressed.

**FIGURE 8:**
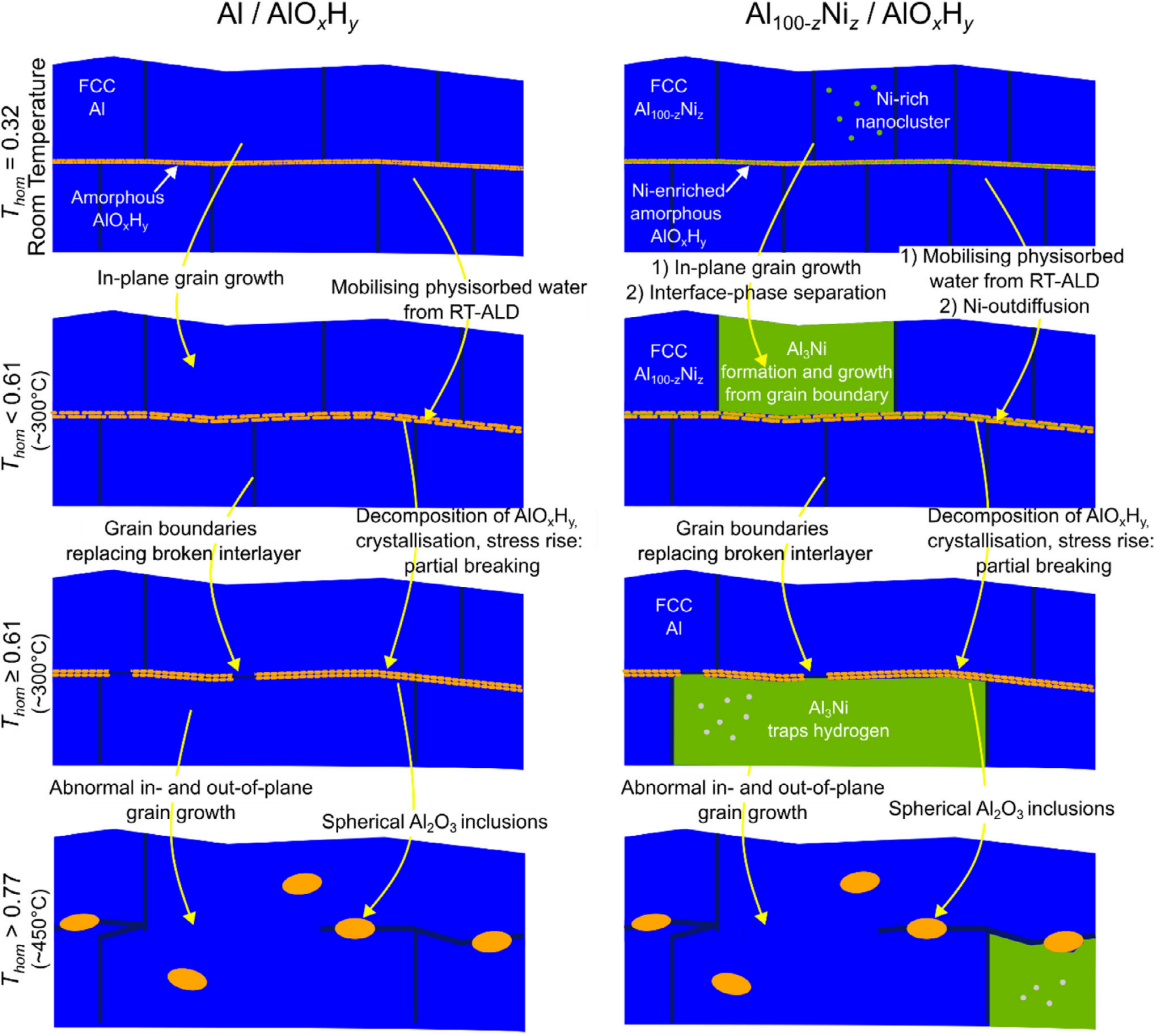
Schematic illustration of the derived thermal instability mechanisms for Al_100‐_ *_z_* Ni*_z_* / AlO*_x_* H*_y_* nanolaminates, with and without Ni‐addition in the PVD layers, depending on *T_hom_* referencing to Al melting point around of ∼660°C.

### 2.5 Consequences for Mechanical Properties

Ultimately, the evolution of chemistry and microstructure with annealing temperature is put into perspective with the mechanical properties of the Al_100‐_ *_z_* Ni*_z_* / AlO*_x_* H*_y_* thin films to evaluate their thermomechanical robustness. Here, nanoindentation hardness serves as a screening parameter and is displayed as a function of homologous annealing temperature in Figure 9. Due to its nanocomposite nature, a double abscissa is employed for (1) the already established *T_hom_* (*T*/*T_m_*) respecting the Al metal character as well as (2) relative decomposition temperature for the AlO*_x_* H*_y_* interlayers (*T*/*T_decomp._*) assuming AlO*_x_* H*_y_* decomposition around 300°C [62].

**FIGURE 9:**
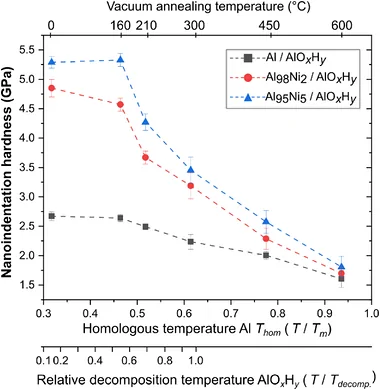
Ex situ nanoindentation hardness of the Al_100‐_ *_z_* Ni*_z_* / AlO*_x_* H*_y_* thin films as a function of homologous temperature / vacuum annealing temperature for 1 h.

Based on the data shown in Figure 9, vacuum annealing of Al_100‐_ *_z_* Ni*_z_* / AlO*_x_* H*_y_* nanolaminates up to *T_hom_* = 0.94 for 1 h is gradually lowering the nanoindentation hardness of all three architectures. The evident strengthening in the Ni‐doped architectures in the as‐deposited state is continuously reduced upon annealing. Notably, the nanoindentation hardness of as‐deposited Al_95_Ni_5_ / AlO*_x_* H*_y_* film is 98% higher (5.3 ± 0.1 GPa) than the reference Al / AlO*_x_* H*_y_* (2.7 ± 0.1 GPa) and this difference is significantly reduced to only 12.5% after vacuum annealing at *T_hom_* = 0.94 (1.8 ± 0.2 GPa versus 1.6 ± 0.1 GPa). An exeption to the linear hardness decay is the capability of all three architectures to quasi‐maintain hardness after vacuum annealing at *T_hom_* = 0.46. Upon further annealing until *T_hom_* ≤ 0.61, two mechanisms are expected to influence the steep hardness decay concurrently: diffusion and reaction within the Al‐Ni layers, and the simultaneous decomposition of the AlO*_x_* H*_y_* layers at 0.6<*T* / *T_decomp._ <* 1.0. Still, the hardness of Ni‐doped films is continuously exceeding the reference Al / AlO*_x_* H*_y_* hardness up to *T_hom_* = 0.77. Correlation of residual stresses in Figure 6 and nanoindentation hardness in Figure 9 additionally reveals the decay in nanoindentation hardness with a rise in tensile residual stress for all samples. However, the direct effect of residual stress on the measured hardness appears to be limited. All films retain nearly constant hardness after vacuum annealing at *T_hom_* = 0.46 (160°C for 1 h; Figure 9), despite an increase in tensile residual stress of approximately 200 MPa (Figure 6). Assuming negligible microstructural evolution during this annealing step, this indicates that moderate changes in residual stress have only a marginal influence on nanoindentation hardness.

We have previously shown that the strengthening in as‐deposited Ni‐doped Al_100‐_ *_z_* Ni*_z_* / AlO*_x_* H*_y_* is a combined consequence of a rise in (crystalline / amorphous) interfaces, dislocation density, and precipitation strengthening in the crystalline matrix that impede dislocation motion through combined efforts [33]. However, as the microstructures change significantly upon annealing, the mechanisms need to be put into consideration. The following discussion is therefore used to predict validity ranges for the mechanisms considering the change in architecture and chemistry. As for nanolaminated materials, yield stress as a function of effective layer thickness (IP grain size x √3  [28]) is displayed in Figure 10. It is explicitly emphasised that the yield stress here is derived from nanoindentation hardness with a Tabor factor of 3 for a facilitated comparison of strength contributions. It is annotated that this relation and therefore the following discussion involves simplification, and the comparisons should be regarded as semi‐quantitative estimates rather than precise quantitative validation. The yield stress as a function of effective Al grain size will then allow a more direct comparison of varying strength upon changing the architecture and chemistry at comparable Al grain size. Here, we propose that, as the effective grain size approaches ∼0.1 (∼100 nm) after annealing at *T_hom_ >* 0.61 (300°C), concurrent with interlayer rupture, the dominant deformation mechanism transitions from confined layer slip to precipitation‐/inclusion‐mediated strengthening. This transition is a mechanistic interpretation due to significant strength drop and the (partial) lack of reinforcing interlayers, rather than a direct experimental verification.

**FIGURE 10:**
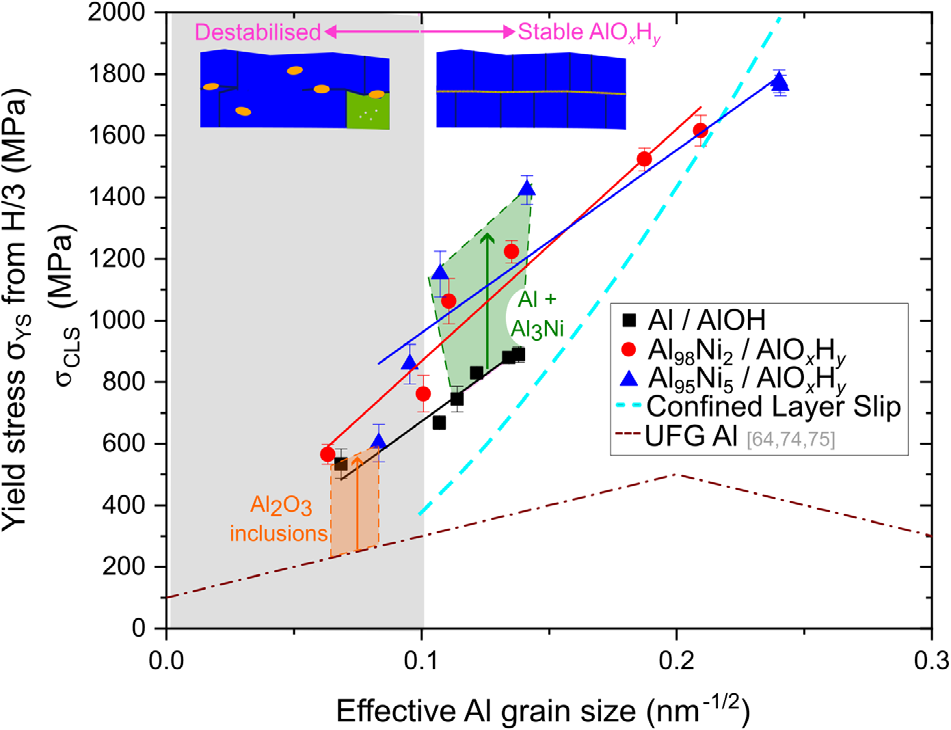
Yield stress *σ_YS_* from nanoindentation hardness H (YS ∼H/3) of the Al_100‐_ *_z_* Ni*_z_* / AlO*_x_* H*_y_* as a function of effective Al grain size (IP grain size x √3  [28]) in a Hall‐Petch plot. Additionally visualised are confined layer slip strengthening [73] (in turquoise) from the respective FCC Al grain size regimes and experimentally observed yield stress for ultrafine‐grained (UFG) Al including the inverse Hall‐Petch regime [64, 74, 75] (in brown). Relevant strengthening regimes stem from confined layer slip (stable AlO*_x_* H*_y_*) [73], Al + Al_3_Ni rule of mixture, and Orowan bowing [76] around spherical Al_2_O_3_ inclusions.

Here, Hall‐Petch coefficient around 6.0 × 10^3^ MPa nm^−1/2^, 7.5×10^3^ MPa nm^−1/2^, and 5.9×10^3^ MPa nm^−1/2^ can be derived for Al / AlO*_x_* H*_y_*, Al_98_Ni_2_ / AlO*_x_* H*_y_*, and Al_95_Ni_5_ / AlO*_x_* H*_y_*, respectively.

Upon annealing, the Al grains are growing and the confined layer slip (CLS) strengthening [73] is therefore progressively decreasing. Simultaneously, but not annotated here, replacing the crystalline / amorphous interfaces through crystalline / crystalline interfaces should reduce strength as reported for Cu / CuZr nanolaminates due to the replacement of strain‐compatible amorphous interlayers [77]. When comparing to nanocrystalline Al in the grain size regime < 0.2 nm^−1/2^ and ∼ 200–500 MPa [64, 74, 75], the derived yield stress for Al / AlO*_x_* H*_y_* of 534–890 MPa is in good agreement with strengthening from CLS, confirming mainly architectural strengthening.

In Ni‐containing films, the softening upon annealing due to Al grain growth is partially compensated by the formation of Al_3_Ni and grain growth. Zhang et al. [78] reported 15% to 19% strengthening from the formation of 100–150 nm Al_3_Ni nanoparticles in the matrix of friction‐stirred Al7075. Similarly, the phase‐separated PVD layers should gain from Al + Al_3_Ni rule‐of‐mixtures strengthening *σ_ROM_* according to:

(2) $$\sigma_{ROM}=V_{Al}\sigma_{Al}+V_{Al_{3}Ni}\sigma_{Al_{3}Ni}$$

with *V_Al_* volume fraction of Ni‐deficient Al, *σ_Al_* yield stress of the Ni‐deficient Al, and *V_Al3Ni_* volume fraction of Al_3_Ni, and *σ_Al3Ni_* yield stress of Al_3_Ni. At effective Al grain size 0.10 nm^−1/2^ < d < 0.14 nm^−1/2^, the Al_95_Ni_5_ / AlO*_x_* H*_y_* has a 54% to 62% higher derived yield stress than the reference Al / AlO*_x_* H*_y_* (Figure 10).

At *T_hom_* > 0.77 (∼450°C), and effective grain size < 0.10 nm^−1/2^ in Figure 10, the CLS model is not applicable anymore due to breaking of the AlO*_x_* H*_y_* interlayers. This is confirmed by the strongly reduced yield stress of Ni‐containing films such that all Al / AlO*_x_* H*_y_*, Al_98_Ni_2_ / AlO*_x_* H*_y_*, and Al_95_Ni_5_ / AlO*_x_* H*_y_* possess a similar yield stress of 567 ± 28 MPa (average Al_100‐_ *_z_* Ni*_z_* / Al_2_O_3_). Here, the growth of Al_3_Ni is not contributing to any strengthening, which is mainly expected from the thin film architecture. Rather, the formation of partially crystalline, spherical *κ*‐Al_2_O_3_ inclusions in the Al_100‐_ *_z_* Ni*_z_* matrix at 600°C induces strengthening. Strengthening in the range of 10% to 170 MPa was also observed in accumulative roll‐bonded ultrafine‐grained (UFG) Al with 0.5 vol.% Al_2_O_3_ particles that additionally induced grain refinement [79]. Here, the Al_100‐_ *_z_* Ni*_z_* / Al_2_O_3_ film after vacuum annealing at *T_hom_* = 0.94 (∼600°C) has a yield stress ∼567 ± 28 MPa (average Al_100‐_ *_z_* Ni*_z_* / Al_2_O_3_) at effective Al grain size in the range of 150–200 nm. At that respective grain size range, pure Al is expected to have a yield stress of 280–400 MPa [64, 74, 75]. Taking the spherical Al_2_O_3_ as obstacles for Orowan bowing into account, strengthening *σ_Bow_* arises from the following equation [76]:

(3) $$\sigma_{\mathrm{Bow}}=0.4M\frac{Gb}{\pi \chi }ln\left(\frac{2r_{s}}{b}\right){\left(1-\nu \right)}^{-0.5}$$

with Taylor factor *M*, shear modulus G, Burger's vector *b*, Poisson's ratio ν, intersection radius of particles *r_s_*, and derived inter‐particle spacing *χ* derived from volume fraction *f* as in [80]:

(4) $$\chi =r_{s}\left(\sqrt{\pi /f}-2\right)$$

Applying an average diameter of 18 ± 7 nm and volume fraction of 0.68% for spherical Al_2_O_3_ inclusions, derived from STEM images in Figure 7, Orowan strengthening in the range of *σ_Bow_* 90–170 MPa has to be expected. Hence, the strengthening by Orowan bowing is in good agreement with the yield stress difference observed for Al_100‐_ *_z_* Ni*_z_* / AlO*_x_* H*_y_* (567 ± 28 MPa) compared to nanocrystalline Al in the same grain size regime (400 MPa [64]).

Overall, the discussion on softening upon annealing Al_100‐_ *_z_* Ni*_z_* / AlO*_x_* H*_y_* demonstrates that the mechanical evolution of the architectures can be quantitatively tracked through the mechanisms of CLS, rule‐of‐mixture strengthening, and Orowan strengthening. Here, the combined framework of synchrotron XRD, S/TEM and APT enables tracking of the microstructural evolution. In Ni‐doped films, the formation and growth of Al_3_Ni grains not only pose obstacles to Al grain growth at low *T_hom_*, but induce significant strengthening too. However, the most significant strengthening can be derived from crystalline / amorphous interlayers due to the significant reduction in yield stress upon destabilisation and interlayer breaking.

## 3 Conclusions

A systematic approach was presented to unravel the thermal (in)stability of Al_100‐_ *_z_* Ni*_z_* / AlO*_x_* H*_y_* nanolaminates by cross‐correlating (synchrotron) XRD, high‐resolution microscopy, and mechanical testing. Following a 1 h vacuum‐annealing treatment at five homologous temperatures from 0.46 to 0.94 (∼160°C to ∼600°C), the results can be summarised in the following manner:

- Synchrotron XRD allowed a non‐destructive, reliable, and efficient investigation of angle‐dependent FCC Al crystallite size (out‐of‐plane and in‐plane).
- At low homologous temperatures (*T_hom_* ≤ 0.52, ∼210°C), two competing mechanisms dominate the microstructure evolution: (1) Ni‐segregation at Al grain boundaries and (2) phase separation FCC Al_100‐_ *_z_* Ni*_z_* → FCC Al and orthorhombic Al_3_Ni. This phase separation effectively limits the thermal stability, reducing the driving force for stabilising Al grain boundaries.
- Hydroxylated 1 nm ALD‐AlO_x_H_y_ interlayers deposited at room temperature decompose upon annealing; the combined analytical framework suggests both interlayer swelling and oxidation of the adjacent Al layers. Crystallisation to κ‐Al_2_O_3_ and subsequent interlayer breaking are observed with an onset below T_hom_ = 0.77 (∼450°C). Together, these findings suggest that the hydrogen impurities in the ALD layers promote out‐of‐plane Al grain growth occurring at lower temperatures than expected.
- Upon further annealing at *T_hom_* = 0.94 (∼600°C), the nanolaminated architecture is fully dissolved and partially crystalline, spherical Al_2_O_3_ particles embedded in the coarser grained Al(_100‐_ *_z_* Ni*_z_*) matrix are found.
- Combined, the presented degradation mechanisms cause a continuous decay in nanoindentation hardness. While at (close‐to) ambient conditions, chemistry variation from Al_100‐_ *_z_* Ni*_z_* significantly influences hardness, at higher temperatures (*T_hom_* > 0.77), the dominant mechanisms change, and the hardness is controlled primarily by the cooperative effects between the alloy matrix and the oxide precipitates.

Overall, the combined structural and mechanical analyses reveal that the thermal instability of Al_100‐_ *_z_* Ni*_z_* / AlO*_x_* H*_y_* nanolaminates is governed by a sequence of coupled phase separation and decomposition processes that progressively dissolve the nanoscale architecture. Future work toward more stable PVD‐Al*Q* (*Q* dopant elements) / ALD‐Al_2_O_3_ nanolaminates should therefore focus on applying (1) Al‐*Q* layers without matrix precipitation and crystalline Al_2_O_3_ stabilisation as well as (2) altered ALD‐Al_2_O_3_ processing to reduce the water content (hydroxylated species). It is emphasised that great potential is seen here for ALD‐Al_2_O_3_ from O_3_ / O‐plasma and TMA, while high temperature ALD is limited by significant coarsening of the nanocrystalline Al grains during deposition. Finally, the here presented findings establish a comprehensive framework to correlate atomic‐scale transformations with macroscopic property degradation in complex metal/oxide nanocomposites, which is of interest for a broad range of laminated coatings such as emerging H barrier coatings.

## 4 Experimental Section

The thermal stability of nanolaminated Al_100‐_ *_z_* Ni*_z_* / AlO*_x_* H*_y_* was investigated after 1 h vacuum annealing over a homologous temperature range of *T_hom_* = 0.46–0.94 (≈160–600°C) using a correlative multi‐scale characterisation approach combining (synchrotron) XRD, HR‐S/TEM, and atom probe tomography (APT). Orientation‐resolved synchrotron XRD provided statistically robust grain‐size analysis over macroscopic sample areas, complementing nanoscale structural and compositional characterization to establish processing–microstructure–property relationships. This integrated framework reveals how nanolamination, solute segregation, and interfacial transformations control microstructural evolution and the retention/loss of mechanical strengthening during thermal exposure, providing design insights for improving the thermal stability of PVD/ALD nanocomposites.

The thin films were deposited by means of hybrid PVD (Al_100‐_ *_z_* Ni*_z_*) and ALD (AlO*_x_* H*_y_*) in a SwissCluster AG SC‐1. Al_100‐_ *_z_* Ni*_z_* layers were deposited from two elemental Al targets and one Ni target at room temperature, while AlO*_x_* H*_y_* was grown by room‐temperature ALD from trimethylaluminium (TMA) and deionised (DI)‐H_2_O. The metallic Al_100‐_ *_z_* Ni*_z_* layers were deposited by physical vapour deposition (PVD) under controlled vacuum conditions: the base and working pressure were always < 9×10^−7^ and ∼5×10^−3 ^mbar. Applying 2×280 mA direct current magnetron sputtering on 2“ Al (99.999%, HMW Hauner AG, Germany) and 1×15‐33 mA high power impulse magnetron sputtering on 2” Ni (99.999%, HMW Hauner AG, Germany), the resulting deposition rate was ∼0.08 nm s^−1^. All films were deposited on Si+80 nm Si_3_N_4_ (Microchemicals AG, Germany) substrates without rotation. Prior to this, film thicknesses and chemistries were calibrated by depositing monolithic Al_100‐_ *_z_* Ni*_z_* on TEM membranes by varying deposition time and magnetron power densities. The AlO*_x_* H*_y_* interlayers were subsequently introduced by room‐temperature ALD using TMA and DI‐H_2_O with defined pulse/purge sequences: TMA pulse of 200 ms at 250 sccm Ar, and purge of 20 000 ms at 500 sccm Ar; DI H_2_O pulse of 150 ms at 250 sccm, and purge of 20000 ms at 500 sccm Ar. An oxide growth per cycle of around 0.14 nm cycle^−1^ was reached. The multilayer architecture consisted of 114 Al_100‐_ *_z_* Ni*_z_* – AlO*_x_* H*_y_* bilayers.

An Advance Rico Mini Lamp Annealer MILA‐5050 under continuous Ar flow of 1000 sccm and vacuum backpressure around 5×10^−2^ mbar at heat ramping of 10 K min^−1^ was utilised for the annealing experiments at 160°C, 210°C, 300°C, 450°C, and 600°C for 1 h (in the manuscript, referred to as vacuum annealing). While the residual oxygen/water partial pressure was not directly monitored but affect the interfacial chemistry, the reduced backpressure combined with continuous Ar flow is expected to substantially limit residual oxidizing species compared to ambient conditions. The lower homologous temperatures *T*_hom_ = 0.46 (∼160°C) and *T*_hom_ = 0.52 (∼210°C) were chosen to trigger mainly Ni diffusion in the Al matrix, while keeping the nanolaminated structure stable, similarly to previous studies on doped nanocrystalline Al [45]. The higher temperatures *T_hom_* = 0.61, 0.77, and 0.94 (∼300°C, ∼450°C, and ∼600°C) allow investigation of microstructure evolution under harsher conditions and screening of potential application temperatures. It is emphasised that all the samples were isothermally annealed without prior thermal treatment, and the homologous temperature was derived from *T_m_* = 933 K (660°C) to allow comparison to Al alloys.

For analysis, we combine (synchrotron) XRD, S/TEM, and APT to complementary track the phase and chemistry evolution in Al_100‐_ *_z_* Ni*_z_* – AlO*_x_* H*_y_*. Synchrotron XRD is a powerful technique to track the angle‐dependent evolution of coherent (111) FCC Al crystallite size, residual stress, and crystallisation of AlO*_x_* H*_y_*. S/TEM adds information on the local phase evolution and chemistry, as well as interface segregation tendencies, while APT provides information on the diffusion behaviour of the (light) elements.

XRD patterns of Al – AlO*_x_* H*_y_*, Al_98_Ni_2_ – AlO*_x_* H*_y_*, and Al_95_Ni_5_ – AlO*_x_* H*_y_* thin films were acquired directly after the samples were taken out of the vacuum furnace in a Bruker D8 Discovery in 2*θ*‐range from 20 to 100° at 40 kV and 40 mA. The instrument broadening was corrected by measuring a LaB_6_ standard.

Synchrotron XRD was applied to approach both out‐of‐plane and in‐plane (111) FCC Al coherent crystallite size (in the following OP and IP crystallite size) analysis for each sample. Eight psi angles from *ψ* = 0 up to 70° were chosen to approach OP and almost IP (111) FCC Al crystallite size. Three phi angles (*Φ* = 0, 45, and 90°) were chosen for comparability. A wavelength of 0.128 nm was used, and the instrument broadening was corrected by measuring a LaB_6_ standard at every angle *ψ*. Ultimately, the *ψ* ‐dependent (111) FCC lower‐bound coherent domain size of each sample was analysed by applying the Scherrer equation with a shape factor equal to 1, allowing the best fit to as‐deposited layer thickness of ∼25 nm under the assumption of equiaxed grains. Here, the Scherrer lower‐bound coherent domain size for the IP condition is termed “crystallite size” as a short‐hand in the following. From the same dataset, sin^2^ *ψ* analysis [81] was performed to determine the residual stress and stress‐free lattice parameter evolution over chemistry and temperature variation.

TEM samples were prepared using a Thermo Fisher Scientific Helios 5 Hydra DualBeam pFIB – scanning electron microscope (SEM) equipped for Xe^+^ milling, and their crystallography and chemistry were subsequently analysed by S/TEM alike the procedure described in [44]. TEM was conducted on a Thermo Fisher aberration corrected (probe) Themis 200 G3 operated at 200 kV. Analyses of S/TEM images, fast fourier transform (FFT) images, and selected area electron diffraction (SAED) patterns were conducted using the CrysTBox software [82]. STEM‐EDS maps were acquired with 20 eV dispersion and 5 µs dwell time and absorption‐corrected assuming ∼100 nm sample thickness and 2.7 g cm^−3^ density.

Atom probe specimens were also prepared in a Thermo Fisher Helios 5 Hydra UX dual‐beam microscope with a pFIB. Xe ions were used at 12 kV for deposition of a protection layer, using a precursor mixture of 99% Pt and 1% C, while milling operations were conducted at 30 kV. Plan‐view lift‐outs were done (see supplementary material of [83]) in order to align the Al_100‐_ *_z_* Ni*_z_* – AlO*_x_* H*_y_* interfaces parallel to the specimen shank. Sharpening was carried out at 12 kV with the final step at 5 kV. APT was done in a CAMECA local electrode atom probe (LEAP) 4000X HR. Field evaporation was assisted by voltage pulsing with 20% pulse fraction, 100 kHz pulse frequency, 60 K base temperature and 0.5% detection rate. Up to 50 million ions were acquired and data reconstruction was done with CAMECA AP Suite 6.1, employing the shank angle protocol. Visualisation of the interfaces was realised with 10‐nm‐thin slices of the reconstructions. H was included in the ranging of mass spectra and the content can be understood as an upper boundary value due to the likely contribution of residual gas from the analysis chamber. Moreover, C and N impurities with the sum < 1 at.% were detected and are not discussed further.

To track the water precursor adsorption during room‐temperature ALD, in situ quartz crystal microbalance (QCM) monitoring was performed in an SC‐Qube ALD system (Swiss Cluster) using a 6 MHz RC‐cut quartz crystal with mass changes (∆m/A, in ng/cm^2^) calculated from frequency shifts via Sauerbrey's equation:

(5) $$\Delta f=-\frac{2f_{0}^{2}}{A\sqrt{\rho_{q}\mu_{q}}}\Delta m$$

where *f*_0_ is the initial resonance frequency (6 MHz), *A* is the piezoelectrically active crystal area (in cm^2^), ρ_*q*_ is the quartz density (2.65 g cm^−3^) and μ_*q*_ is the shear modulus of quartz for RC‐cut crystal (2.947·10^11^ g cm^−1^·s^−2^). A conservative relative uncertainty of ±3% was applied to all QCM mass measurements to account for instrumental noise, thermal drift, and long‐term baseline fluctuations.

Nanoindentation was performed on a Zwick Roell nanoindenter system (ZHN Nanoindenter with a 2 N measuring head, ZwickRoell GmbH & Co. KG, Ulm, Germany) equipped with a Berkovich diamond tip. A total of 25 indents were collected per sample condition. The indentation experiments were conducted in a quasi‐continuous stiffness measurement performed in load‐controlled mode with maximum indentation depth of 1 µm. A detailed description of the measurement procedure, including the tip area function and instrument compliance calibration, is provided in [84]. Indentations were conducted with a Berkovich tip (Synton‐MDP Ltd., Switzerland) up to a maximum load of 100 mN. Depth‐resolved hardness (*H*) profiles were extracted from the load‐displacement curves according to the Oliver and Pharr method [85]. The film‐only hardness values were derived from a plateau region between 100–300 nm indentation depth as proposed by Fischer‐Cripps [86] to minimise substrate effects. After discarding outliers, at least 20 valid indents were collected for each condition, and the measurement error is derived from indent‐to‐indent variation and indent‐over‐depth variation. It is annotated that slight pile‐up from plastic behaviour was observed for every sample.

## Author Contributions

**H.C. Jansen**: Investigation, Writing – original draft, Writing – review & editing, Conceptualisation, Methodology. **B. Putz**: Investigation, Writing – review & editing, Validation, Funding Acquisition. **A. Sharma**: Investigation, Writing – review & editing. **L. Lapeyre**: Investigation. **F.F. Klimashin**: Investigation, Writing – review & editing. **D. Gutnik**: Investigation. **M. Hans**: Investigation, Visualisation, Writing – review & editing. **J.M. Schneider**: Writing – review & editing, Resources, Funding Acquisition. **J.J. Schwiedrzik**: Writing – review & editing, Supervision, Funding Acquisition. **T.E.J. Edwards**: Writing – review & editing, Conceptualisation, Methodology, Investigation, Validation, Supervision. **J. Michler**: Writing – review & editing, Conceptualisation, Supervision, Resources, Funding Acquisition.

## Conflicts of Interest

The authors declare no conflicts of interest.

## Supporting information




**Supporting File**: smll75393‐sup‐0001‐SuppMat.docx.

## Data Availability

The data that support the findings of this study are available from the corresponding author upon reasonable request.
