# Supplementary material for: Thermal Instability Pathways of Aluminum‐Based Nanocomposites: The Role of Alloying‐Element‐Driven Precipitation and Amorphous Interlayers
Source: Small. 2026 Aug 22;22(60):e75393. doi: 10.1002/smll.75393 (PMC13711475; doi:10.1002/smll.75393)
Supplement: Supplementary file 1 [file SMLL-22-e75393-s001.docx]

**Supplementary Material**

**Thermal Instability Pathways of Aluminum-Based Nanocomposites:**

**The Role of Alloying-Element-Driven Precipitation and Amorphous Interlayers**

***Hendrik C Jansen*** *^a,b^, Amit Sharma ^a^ , Marcus Hans ^c^_,_*

*Fedor F Klimashin ^a^, Léo Lapeyre ^a^, Dominik Gutnik ^d^, Jochen M Schneider ^c^,*

*Thomas EJ Edwards ^a,e,f^* *Jakob Schwiedrzik ^a,g^, Barbara Putz ^a,d^, and Johann Michler ^a,b^*

# S1: Evaluation of in-plane crystallite size (Scherrer lower bound in-plane crystallite size) determination using the angle-dependent synchrotron XRD compared to selected DF-TEM analysis. Note that the following samples were chosen for determination: as-deposited Al / AlO*_x_*H*_y,_* as-deposited Al_98_Ni_2_ / AlO*_x_*H*_y_*, as-deposited Al_95_Ni_5_ / AlO*_x_*H*_y_*, *T_hom_* = 0.52 (~210°C) Al_95_Ni_5_ / AlO*_x_*H*_y_*, *T_hom_* = 0.77 (~450°C) Al_98_Ni_2_ / AlO*_x_*H*_y_*, and *T_hom_* = 0.77 (~450°C) Al_95_Ni_5_ / AlO*_x_*H*_y_* (R^2^=0.89).

# S2: Discussion of activation energy for grain growth of Al_100-_*_z_*Ni*_z_* / AlO*_x_*H*_y_* from the crystallite sizes measured from synchrotron XRD.

Consequently, activation energy analysis of grain growth was approached to allow comparison to conventional nanocrystalline Al (alloys) based on the following equations [1].

$$D_{T}^{1/n}- D_{0}^{1/n}=K \times t$$

(1)

$$\ln K=\ln K_{0}- \frac{Q}{RT}$$

**(2)**

with *D_T_* grain size at temperature *T*, *D_0_* as-deposited grain size, *n* exponent of grain growth, *K* constant, *t* annealing time (3600 s), and *Q* activation energy for grain growth. It is annotate that this simplified model does not take pinning effects into account and is therefore applied only as an approximation [2].

While the chemical complexity and thin-film geometry of the nanocomposites here challenge this model designed for pure metals, a simplified analytical framework effectively extracts the underlying diffusion kinetics. Furthermore, the lack of a detectable maximum grain size within the experimental time-temperature range precludes the use of kinetic equations that account for pinning-induced stagnation. Note that the IP crystallite size from synchrotron XRD was chosen for every temperature as an approximation for the grain size. It is noted that a temperature-dependence of *n* due to abnormal grain growth for nanocrystalline Al exceeding *T_hom_* > 0.77 (~450°C) is expected [3] and two different ranges of *n* are considered: 0.04 ≤ *n* ≤ 0.08 for *T_hom_* ≤ 0.77 (lower boundary *n*) and 0.1 ≤ *n* ≤ 0.28 for *T_hom_* > 0.77 (upper boundary *n*)*.* Respective Arrhenius plots as well as information on activation energies for the full temperature range are displayed in the Supplementary Material S6. Accordingly, Table 1 displays activation energies derived from lower and upper boundary 0.04 ≤ *n* ≤ 0.08 from 23°C ≤ T ≤ 450°C.

Table 1: Activation energies of FCC Al grain growth in nanolaminated Al_100-_*_z_*Ni*_z_* / AlO*_x_*H*_y_*. Lower and upper boundary *n* relate to 0.04 ≤ *n* ≤ 0.08 and include only *T* ≤ 450°C.

|  | Al / AlO*_x_*H*_y_* | | Al_98_Ni_2_ / AlO*_x_*H*_y_* | | Al_95_Ni_5_ / AlO*_x_*H*_y_* | |
| --- | --- | --- | --- | --- | --- | --- |
| Range of grain growth exponent *n*  (according to [3]) | 0.04 | 0.08 | 0.04 | 0.08 | 0.04 | 0.08 |
| Activation energy *Q*  [kJ mol^-1^]  (23°C ≤ *T* ≤ 450°C) | 100 | 54 | 268 | 111 | 374 | 200 |

Here, the activation energy of grain growth can be estimated to be 54 kJ mol^-1^ ≤ *Q* ≤ 100 kJ mol^-1^, 111 kJ mol^-1^ ≤ *Q* ≤ 268 kJ mol^-1^, and 200 kJ mol^-1^ ≤ *Q* ≤ 374 kJ mol^-1^ for Al / AlO*_x_*H*_y_*, Al_98_Ni_2_ / AlO*_x_*H*_y_*, and Al_95_Ni_5_ / AlO*_x_*H*_y_*, respectively. The steep increase of crystallite size from *T_hom_* = 0.77 to 0.94 (~450°C to ~600°C), as well as the recommended adaptation of *n* lead to unrealistic activation energy calculation of grain growth in the range of *Q* > 860 kJ / mol. The activation energies of Al_100-_*_z_*Ni*_z_* / AlO*_x_*H*_y_* therefore partially lie in between reported values for both volume and grain boundary diffusion in nanocrystalline Al with around 197 kJ mol^-1^ and 84 kJ mol^-1^, respectively [4]. The formation of Al_3_Ni as well as the presence of AlO*_x_*H*_y_* - Al_2_O_3_ - interfaces indicate further diffusion than just Al volume and self-diffusion. Especially the low density of ALD-AlO*_x_*H*_y_* compared to ALD-Al_2_O_3_ [5,6] provides accelerated out-of-plane diffusion pathways that can dominate transport kinetics.

However, increasing activation energy upon Ni addition is a hint towards enhanced thermal stability of Ni-doped systems, which can be a result of solute drag, grain boundary segregation, Zener pinning, and/or chemical ordering [7] as discussed in the following. Zhou, Lee, and Lavernia [3] reported an activation energy of grain growth of 79 kJ mol^-1^ for 26 nm Al up to *T_hom_* = 0.83 (~500°C) and 112 kJ mol^-1^ at higher temperatures. Notably, 500°C was the temperature range where stabilisation of 60 nm Al was observed due to Zener pinning by Al_2_O_3_ and Al_4_C_3_ impurities. Tellkamp et al. [8] derived an activation energy of 143 kJ mol^-1^ for an as-milled 16 nm 5083 Al-Mg alloy at *T_hom_* = 0.72 (~400°C), which is consistent with the dominant mechanism of Al self-diffusion at activation energy of 142 kJ mol^-1^ [9].

Arrhenius plots for determination of activation energies of grain growth for Al_100-_*_z_*Ni*_z_* / AlO*_x_*H*_y_*. Lower and upper boundary *n* relate to 0.04 ≤ *n* ≤ 0.08 for *T_hom_* ≤ 0.77 (~450°C) and 0.10 ≤ *n* ≤ 0.28 for *T_hom_* > 0.77. Note that the derived activation energies strongly depend on the respected grain size when (a-c) including or (d-f) excluding Al_100-_*_z_*Ni*_z_* / AlO*_x_*H*_y_* grain size determined from synchrotron XRD after vacuum annealing at *T_hom_* = 0.94 (~600°C).

# S3: Grain size analysis including dark field (DF) – transmission electron microscopy (TEM) images with objective aperture covering (111) and (200) FCC Al reflections for selected thin films as-deposited (a) Al / AlO*_x_*H*_y_* ,(b) Al_98_Ni_2_ / AlO*_x_*H*_y_*, and (c) Al_95_Ni_5_ / AlO*_x_*H*_y_*; *T_hom_* = 0.52 (~210°C) (d) Al_95_Ni_5_ / AlO*_x_*H*_y_*; and *T_hom_* = 0.77 (~450°C) (e) Al_98_Ni_2_ / AlO*_x_*H*_y_* and (f) Al_95_Ni_5_ / AlO*_x_*H*_y_* including hence-measured IP Al grain size statistics. Note that scale bars differ, while always the same abscissae scaling was used.

# S4: APT reconstructions of atomic positions (10 nm slice), (a), including Al, Ni, O, and H composition profiles for Al_98_Ni_2_ / AlO*_x_*H*_y_* at *T_hom_* = 0.52 (~210°C), (b-c), as well as the respective mass-spectrum, (d).

3

# S5: APT mass spectra for Al_95_Ni_5_ / AlO*_x_*H*_y_* (a) *T_hom_* = 0.32 (as-deposited, adapted from [68]), (b) *T_hom_* = 0.52 (~210°C), and (c) *T_hom_* = 0.77 (~450°C).

# S6: S/TEM results on Al_98_Ni_2_ / AlO*_x_*H*_y_* after annealing at *T_hom_* = 0.77 (~450°C): (a) HAADF-yield with SAED pattern, (b) Al-O-Ni-yield.

# S7: Representative quartz crystal microbalance (QCM) signal recorded over three consecutive TMA-H_2_O ALD cycles performed at room temperature (RT). The QCM sensor was pre-coated with a 50 nm ALD-grown Al_2_O_3_ layer to ensure a consistent and hydroxylated surface prior to measurement. A characteristic mass gain of 9.3 ± 0.2 ng.cm^-2^ is observed during the H_2_O half-cycle, attributed to a water tank effect from the Al_2_O_3_ layer. This value was obtained by averaging 10 ALD cycles, with uncertainty representing the standard deviation across the cycles. Each cycle consisted of a 0.15 s TMA pulse, 20 s Ar purge, 0.15 s H_2_O pulse and 20 s Ar purge.

# S8: Surface secondary electron SEM images of Al_100-_*_z_*Ni*_z_* / AlO*_x_*H*_y_* after vacuum annealing to visualise surface artefacts.

# S9: Nanoindentation (a) hardness and (b) Youngs modulus [E] of Al_100-_*_z_*Ni*_z_* / AlO*_x_*H*_y_* as a function of penetration depth.

# References

[1]	J.E. Burke, D. Turnbull, Recrystallization and grain growth, Prog. Met. Phys. 3 (1952).

[2]	F. Liu, R. Kirchheim, Nano-scale grain growth inhibited by reducing grain boundary energy through solute segregation, J. Cryst. Growth 264 (2004) 385–391. https://doi.org/10.1016/j.jcrysgro.2003.12.021.

[3]	F. Zhou, J. Lee, E.J. Lavernia, Grain growth kinetics of a mechanically milled nanocrystalline Al, Scr. Mater. 44 (2001) 2013–2017. https://doi.org/10.1016/S1359-6462(01)00826-0.

[4]	A. Jankowski, J. Ferreira, J. Hayes, Activation energies of grain growth mechanisms in aluminum coatings, 491 (2005) 61–65.

[5]	C. Guerra-Nuñez, M. Döbeli, J. Michler, I. Utke, Reaction and Growth Mechanisms in Al2O3 deposited via Atomic Layer Deposition: Elucidating the Hydrogen Source, Chem. Mater. 29 (2017) 8690–8703.

[6]	C. Cancellieri, S. Gramatte, O. Politano, L. Lapeyre, F.F. Klimashin, K. Mackosz, I. Utke, Z. Novotny, A.M. Müller, C. Vockenhuber, V. Turlo, L.P.H. Jeurgens, Effect of hydrogen on the chemical state, stoichiometry and density of amorphous Al2O3 films grown by thermal atomic layer deposition, Surf. Interface Anal. 56 (2024) 293–304.

[7]	T.R. Malow, C.C. Koch, Grain growth in nanocrystalline iron prepared by mechanical attrition, Acta Mater. 45 (1997) 2177–2186.

[8]	V.L. Tellkamp, S. Dallek, D. Cheng, E.J. Lavernia, Grain growth behavior of a nanostructured 5083 Al – Mg alloy, J. Mater. Res. 16 (2001).

[9]	Y. Li, S.R. Nutt, F.A. Mohamed, An investigation of creep and substructure formation, Acta Mater. 45 (1997).
